# Metabarcoding with Illumina and Oxford Nanopore Technologies provides complementary insights into tree seed mycobiota

**DOI:** 10.1186/s40793-025-00712-7

**Published:** 2025-05-19

**Authors:** Jana Mittelstrass, Renate Heinzelmann, René Eschen, Martin Hartmann, Quirin Kupper, Salome Schneider, Simone Prospero, Iva Franić

**Affiliations:** 1 Eidgenössische Forschungsanstalt für Wald, Schnee und Landschaft WSL, Birmensdorf, Switzerland; 2 CABI, Delémont, Switzerland; 3 Institute of Agricultural Sciences, ETH Zürich, Zurich, Switzerland

**Keywords:** High-throughput sequencing, Amplicon sequencing, Read length, MinION, ITS, Endophytes, Pathogens, Fungi, Seed mycobiome

## Abstract

**Background:**

Culturing of fungi is labor-intensive and reveals limited diversity, while high-throughput sequencing of barcodes (i.e., metabarcoding) enables a simultaneous detection of fungi from multiple environmental samples. Metabarcoding using short-read sequencers, such as Illumina platforms, provides high sequencing depths but results in many unidentified taxa. Long-read sequencing can improve species and genus assignments but might encompass lower sequencing depth and limit diversity coverage. In this study, fungi in seeds of eleven angiosperm and gymnosperm tree species were assessed using traditional culturing, Illumina short-read metabarcoding, and Oxford Nanopore Technologies long-read metabarcoding. We focused on seed-borne fungi as understanding their diversity and potential impacts on seedlings is crucial for securing plant health. We compared (1) the number and identity of fungal genera and species between metabarcoding approaches and traditional culturing and (2) fungal alpha- and beta-diversity between metabarcoding methods, considering different hosts and fungal lifestyles.

**Results:**

In both short- and long-read metabarcoding datasets, similar numbers of fungal reads and operational taxonomic units were assigned to comparable numbers of fungal genera and species. About one-third of the identified genera were plant pathogens, followed by saprotrophs and endophytes. Culturing overall revealed fewer fungal genera, while most of the fungal reads in short-read metabarcoding datasets stemmed from cultured taxa. Long-read metabarcoding revealed lower per-sample diversity than short-read metabarcoding and distinct fungal communities compared to those from the short-read datasets. Host-dependent patterns in alpha- and beta-diversity were observed across methods, with angiosperms harboring more fungal taxa than gymnosperms, and distinct community structuring across host tree groups and species, although the differences were stronger in short-read than long-read metabarcoding datasets.

**Conclusions:**

Illumina and Oxford Nanopore Technologies metabarcoding captured similar host-dependent diversity patterns despite observed differences in numbers and composition of fungi. Short-read metabarcoding might be optimal for fungal biodiversity studies due to higher sequencing depths and resultant breadth of diversity. As error rates are continuing to decrease, reference databases expand, and throughput improves, long-read metabarcoding is becoming a strong candidate for future diagnostic studies of fungi. Traditional culturing captures most of the fungi from short-read metabarcoding and remains valuable for obtaining isolates for further research.

**Supplementary Information:**

The online version contains supplementary material available at 10.1186/s40793-025-00712-7.

## Background

Plant seeds harbor diverse fungal communities, some inherited from the mother plant and some acquired from the environment [1]. As a result, seed mycobiota (i.e., fungal spores and hyphae inhabiting seeds) exhibit host-driven diversity patterns, determined by host phylogeny and plant traits, and spatio-temporal diversity patterns driven by abiotic factors such as geographic location and climate [2–4]. Tree seed mycobiota, although historically understudied, have been the focus of an increasing number of microbiome studies in recent years. The studies explored the diversity of seed-borne fungi to assess the risks of pathogen movement with tree seed trade [5, 6], to understand the relative importance of the host and environment in shaping diversity of tree seed fungi [3, 7, 8] and to unravel the assembly processes in tree seedlings [9]. Growing interest in tree seed mycobiota is mainly due to the increasingly recognized implications seed-associated organisms have for ecosystem functioning and services, and for commercial forestry [10, 11]. For example, some important tree pathogens are known to be seed-borne [10]. The pathogen *Fusarium circinatum*, the causal agent of pine pitch canker, and *Diplodia sapinea,* an opportunistic fungal pathogen causing Diplodia tip blight, have been linked to the movement of pine seeds with devastating consequences for pine plantations [12–14]. However, not all fungi associated with seeds are detrimental to seed and seedling health and development. In fact, there is growing evidence that seed and seedling development may benefit from some seed fungi [1, 15, 16] through improved seed quality, plant growth promotion and seedling development [17, 18], or increased resistance to biotic and abiotic stresses. Thus, the development of efficient tools to detect and identify seed mycobiota is critical to better understand the potential of these complex communities to reduce or improve plant performance.

Since the second half of the nineteenth century, pro- and eukaryotic microorganisms, including fungi associated with plants, have been studied using culturing methods. In these methods, specific fungi are isolated from their original substrate, grown in pure culture on nutrient media, observed under the microscope, described, and sometimes used for further experiments (e.g., to test interactions with the host or to determine the host range). Using culturing methods, fungi are identified either based on morphological characteristics of their cultures, fruiting bodies, or spores [19, 20], and—since the second half of the twentieth century—by DNA-based methods. The identification of cultures through Sanger sequencing has been widely employed for decades [21]. However, obligate biotrophs, such as powdery mildews, downy mildews and rusts depend on living host cells and thus cannot be cultured [22]. Similarly, isolating rare or slow-growing fungi [21, 23] can be done but requires meticulous culturing work and patience. Some of the limitations associated with culturing techniques can be addressed by using high-throughput sequencing (HTS) technologies.

High-throughput sequencing of molecular marker genes (i.e., metabarcoding) allows a rapid parallel assessment of many individuals, including obligate biotrophs and rare taxa, directly from host tissues. Technological advances [24, 25], population of reference sequence databases [26] and the reduction of sequencing costs [27] have ensured a broad user base for platforms such as Illumina MiSeq, resulting in a myriad of studies revealing unprecedented microbial diversity in various biotic [8, 28–32] and abiotic environments [33, 34]. At the same time, the key challenges associated with metabarcoding include the need for adequate laboratory and analytical infrastructure, particularly bioinformatic analysis [35], and incomplete reference sequence databases for taxonomic identification [36–38]. To ensure amplification of the target taxa, special care must be taken in the selection of appropriate barcoding markers [39, 40], while, in case of plant-associated microbiota, reducing host interference [43].

A major bottleneck of metabarcoding on the Illumina sequencing platform is that it yields short amplicons of up to maximum ~ 550 bases in length using paired-end approaches. The fungal marker of choice, the internal transcribed spacer (ITS) region, spans up to 700 base pairs (bp) in most fungi [41, 42] but shows considerable interspecific size variation of multiple hundred bp [43, 44]. Current Illumina technology only allows the sequencing of one of the ITS subregions (i.e., ITS1 or ITS2). Albeit both ITS subregions are sufficiently variable for a broad range of fungi [41], depending on the primers or ITS region used, taxonomic biases may arise [42]. Thus, targeting the entire ITS at once may provide greater taxonomic resolution [45] and improve species identification. Technological advances are now fuelling the further development of novel long-read sequencing technologies, such as Pacific Biosciences [46] or Oxford Nanopore Technologies (ONT) [47]. ONT sequencing using the MinION platform can be done in-house or even in field settings [48], at economic prices and can deliver reads of tens to hundreds of kilobases [45, 49]. Long sequences can increase chances of correct species identification [50] by capturing more phylogenetic information in longer amplicons, which is also critical for accurate assignment of fungal lifestyles [51]. These novel long-read sequencing technologies have been methodologically validated using bacterial [52] and fungal [36, 53, 54] mock communities with strains expected in the studied environments, and ONT has been applied for the characterization of water- [55] or plant-associated fungi [47, 50, 56], among others. In line with other studies that have compared the influence of methodology on assessed mycobiomes, a comparative study of the ONT and Illumina platform, alongside traditional culturing methods, would provide a comprehensive understanding of differently assessed fungal communities when done across tree species or origins.

The main objective of this study was to systematically compare seed mycobiota of eleven angio- and gymnosperm tree species determined by (i) short-read Illumina metabarcoding using two different primer pairs targeting ITS2, (ii) long-read ONT metabarcoding targeting the full-length ITS region and (iii) traditional fungal culturing with Sanger sequencing, to assess whether different fungal assessment approaches capture fungi with similar taxon and lifestyle assignments, and to determine whether host-dependent diversity patterns are preserved across methods. We hypothesized that long-read sequencing would reveal lower per-sample diversity due to lower sequencing depth but a higher taxonomic resolution resulting in more identified fungal genera and species than short-read sequencing (ONT vs. Illumina). Moreover, we expected culturing to reveal a smaller fraction of the total community than the metabarcoding approaches.

## Methods

### Sample acquisition

The analyzed seed samples were obtained in 2016 [6] and belonged to 58 commercially traded seed lots of five angiosperm and six gymnosperm tree species originating from three continents (Table 1). Fungi were assessed from a total of 100 seeds per seed lot. Seeds were surface-sterilized with sodium hypochlorite (0.5%) for 5 min, subsequently rinsed twice with sterile water and air-dried under sterile conditions [6]. Each seed was cut in half and one half was used for fungal culturing (see below), while the other halves were pooled per seed lot and subjected to DNA extraction using the DNeasy PowerPlant Pro Kit (Qiagen, Hilden, Germany). The DNA was used as a template for Illumina MiSeq sequencing of two different ITS2 amplicons (‘Taylor’ and ‘Tedersoo’) and for ONT MinION sequencing, spanning the whole ITS region (Supplementary Table S1). While the ‘Taylor’ dataset and the culturing data were previously described in Franić et al. [6], the ‘Tedersoo’ and ‘ONT’ datasets were examined together in this study.

**Table 1:** Numbers of seed lots analyzed using each method and their distributions across host tree species, host tree group and their continent of origin

Tree species	Tree group	Origin	Illumina meta-barcoding	ONT meta-barcoding	Culturing
*Acer macrophyllum*	Angiosperm	North America	6	4	5
*Acer palmatum*	Angiosperm	Asia	5	5	5
*Acer pseudoplatanus*	Angiosperm	Europe	3	2	3
*Fagus sylvatica*	Angiosperm	Europe	6	5	5
*Quercus garryana*	Angiosperm	North America	6	5	6
*Larix gmelinii*	Gymnosperm	Asia	3	3	3
*Picea abies*	Gymnosperm	Europe	5	4	4
*Pinus ponderosa*	Gymnosperm	North America	8	7	6
*Pinus sylvestris*	Gymnosperm	Europe	5	3	4
*Pinus tabulaeformis*	Gymnosperm	Asia	4	4	4
*Tsuga heterophylla*	Gymnosperm	North America	7	6	5
*n* = *11*	*n* = *2*	*n* = *3*	*n* = *58*	*n* = *48*	*n* = *50*

### Fungal assessment by metabarcoding

#### Illumina MiSeq sequencing

Two Illumina ITS2 libraries were generated and bioinformatically processed using protocols and methodology previously published by Franić et al. [6]. For library preparation, the DNA of each sample (seed lot) was quantified using the Qubit dsDNA BR Assay Kit (Invitrogen, Thermo Fisher Scientific Inc., Waltham, MA, USA). After dilution to 10 ng/µL, 5 µL of template DNA were amplified in PCR with both primer sets (for primer overview and sequences, see Supplementary Table S1) in triplicates as described in Franić et al. [6]. Briefly, for the Taylor dataset, the primer pair *5.8S-Fun*/*ITS4-Fun* [57] was used in PCR reactions with a total volume of 20 µL using the JumpStart REDTaq ReadyMix Reaction Mix (Sigma Aldrich, St. Louis, MO, USA). PCRs were run on a Veriti 96-Well Thermal Cycler (Applied Biosystems, Thermo Fisher Scientific Inc.) for 2 min at 94 °C; followed by 35 cycles of 30 s at 94 °C, 30 s at 58 °C, and 2 min at 72 °C; and a final extension of 10 min at 72 °C. For the Tedersoo library, PCR was carried out using the forward primer *ITS3ngs-mix* [33, 39] and the reverse primer *ITS4ngsUni* [40] with otherwise equal cycling conditions to those described above, except for an annealing temperature of 55 °C and a final concentration of 0.5 mM MgCl_2_ in the reaction. Success of amplification, and lack of amplification in the negative (non-template) controls, was verified by agarose gel electrophoresis. The PCR products for both Taylor and Tedersoo amplicons were sent for library construction and sequencing to the Génome Québec Innovation Center (McGill University, Montréal, Canada) where all samples were sequenced on an Illumina MiSeq (Illumina Inc., San Diego, CA, USA) instrument using v3 chemistry (2 × 300 bp).

A customized bioinformatic pipeline, which is based on UPARSE [58] and described in detail in Franić et al. [6], was used for processing the demultiplexed files for both datasets. Briefly, the demultiplexed sequences were merged, then, PhiX and primer sequences were filtered out [59, 60]. Sequences were then quality-filtered and denoised, the ITS2 region was extracted using ITSx [61], and resulting sequences were clustered at 98% into unique Operational Taxonomic Units (OTUs) using UNOISE [62]. For the current study, these OTUs were taxonomically (re-)classified with a Naïve-Bayes classifier implemented in QIIME2 (version qiime2-2022.2 [63]) using the fungal UNITE database (version 10.05.21 [38]). To test the proportion of reads originating from the host trees, the UNITE database including other eukaryotic reference sequences (‘All eukaryotes’) was used. The raw sequences of the Tedersoo dataset were deposited at the European Nucleotide Archive (ENA) under accession number PRJEB81281, while raw sequences of the Taylor dataset were deposited in the Sequence Read Archive under accession number PRJNA550270 [6]. To minimize the presence of artefacts due to sequencing errors [49], singletons (i.e., OTUs with one read count) were excluded from analyses, unless stated otherwise. In both Illumina metabarcoding datasets, singletons amounted to below 2.5% of the total reads (i.e., Taylor: 2.2%, Tedersoo: 0.63%).

#### ONT MinION sequencing

From 48 out of 58 DNA extracts produced in Franić et al. [6], libraries of the ribosomal RNA gene (rRNA) were prepared and sequenced on a MinION instrument (Oxford Nanopore Technologies, Oxford, UK), using a FLO-MIN106 flow cell (R9.4.1 pore chemistry). The primers *NS1short/RCA95m* [36] were used for the amplification of a ~ 4-6 kB fragment, including the SSU, the full ITS region and part of the LSU of the rRNA gene (Supplementary Table S1). PCR reactions were run in three technical replicates. For each reaction, 5 µL of DNA template previously diluted to 10 ng/µL was added to the reaction mix. This consisted of 1 × PrimeSTAR GXL Buffer (including 5 mM Mg^2+^), 0.8 mM dNTPs, 0.3 mg/mL BSA (Sigma Aldrich, Steinheim, Germany), 400 nM of each primer, and 0.5 U PrimeSTAR GXL DNA Polymerase (Takara Bio Europe, Saint-Germain-en-Laye, France), which was then filled up to the final reaction volume (20 µL) with PCR-grade water (Merck, Darmstadt, Germany). The following parameters were used to run PCR on a Veriti thermocycler (Applied Biosystems, Waltham, MA, USA): 1 min denaturing at 98 °C, followed by 29 cycles of 98 °C for 10 s, 55 °C for 15 s, 68 °C for 2.5 min. The pooled technical PCR replicates for each sample were visually inspected in gel electrophoresis. For samples that did not produce a visible amplicon band, DNA was diluted 1:20 v/v prior to PCR repetition. Each sample was then cleaned up using AMPure Beads XP (Beckman Coulter, Brea, CA, USA) following the manufacturer’s instructions with small modifications (bead ratio of 0.5 (v/v), 80% ethanol, elution of the DNA after incubation at 37 °C for 15 min).

Unique 24-bp barcodes (Supplementary Table S2, EXP-PBC096 barcoding kit, ONT) were added in a limited-cycle PCR to each sample. The cycling conditions consisted of an initial denaturation step of 1 min at 98 °C, followed by 15 cycles of 98 °C for 10 s, 60 °C for 15 s, 68 °C for 4 min, and a final elongation step at 68 °C for 10 min. From each sample, 7 µL of the cleaned PCR product were used as template in a total reaction volume of 50 µL, including the same cycling conditions described above but using 1 mM dNTP Mix, 1.25U PrimeSTAR GXL DNA Polymerase and 200 nM of the barcoded primer. Resulting barcoded products were cleaned-up with AMPure Beads XP as described above and then pooled equimolarly: ~ 1 µg of the pooled libraries was used as input for the library preparation with the Ligation Sequencing Kit SQK-LSK109 (Oxford Nanopore Technologies, Oxford, UK). Each sequencing run included 12 samples (Supplementary Table S2) and the flow cell used for runs 2 and 3 was washed between runs according to the manufacturer’s instructions.

The software *guppy* (ONT, version 4.5.4) was used for the basecalling and demultiplexing of the raw reads. The numbers of reads per sample after demultiplexing (Supplementary Table S2) yielded a total of 7.98 M sequences. These sequences were further processed on high-performance clusters of the Federal Institute of Technology Zurich (ETH), Switzerland and the Swiss Federal Institute for Forest, Snow and Landscape Research WSL. The reads were re-oriented with *seqtk* (version 1.2-r94; https://github.com/lh3/seqtk.git), and the primers were detected and trimmed with *cutadapt* (version 1.12 [60]) with the error rate parameter -e set to 2. Only the reads in which both the forward and reverse primers were found in the correct orientation were retained. The trimmed reads were then filtered using *prinseq-lite* (version 0.20.4 [64]). Only sequences with lengths > 1 kB and < 8 kB, and GC contents between 40 and 55 were retained.

After these processing steps, the full-length ITS region was extracted from the filtered sequences using *ITSx* (version 1.1.3 [61]). The parameter -E was set to 0.001 and the complement-parameter was set to TRUE. We extracted the ITS region from the ONT sequences to be able to compare the sequences of all datasets among each other, although this meant discarding parts of the LSU and SSU included in the ONT sequence. Although these markers can contribute to the identification of fungi by phylogenetic placement, this approach is not yet commonly used and needs further development [65]. Then, the following steps of ONT data processing were chosen to mimic steps (i.e., clustering and mapping) undertaken in the Illumina data-processing pipeline (see Franić et al. [6]), but also considering the inferior sequence quality (i.e., choosing different parameters) of the ONT dataset due to high error rates in the ONT version used [66, 67]. The filtered, full-length ITS sequences were concatenated and further processed with *vsearch* v2.22.1 [68]. First, the sequences were pre-clustered with the *cluster_size* algorithm using a cluster ID of 0.75 while restricting the minimum and maximum sequence length to 250 bp and 1 kB, respectively. These parameters for sequence length were chosen to minimize presence of host tree sequences. Using the *sortbysize* and *uchime_denovo* algorithms, first global singleton sequences and subsequently chimeras were removed. After relabeling with *fastx_filter* to include the prefix ‘OTU_’, the initial sequences were matched to the OTUs with the *usearch_global* algorithm, resulting in the final OTU table. During this step, the ‘matching id’ parameter was set to 0.97, while ‘maxhits’ was set to 1 and ‘maxaccepts’ to zero. This step was necessary for the generation of the ONT OTU table, but also led to the loss of reads in the ONT dataset: in the ONT dataset, OTUs were almost exclusively singletons due to error rates higher than the clustering threshold, an issue that is known to be associated with the former ONT chemistry (versions on flow cells < R10.0) [66]. Consequently, further ONT analyses were based on all obtained reads per sample without excluding the singletons, as it was done for the Illumina datasets.

Lastly, as described for the Illumina metabarcoding datasets, the sequences of the ONT OTUs were classified against the fungal UNITE database (version 10.05.21 [38]) using a Naïve-Bayes classifier in QIIME2 (version qiime2-2022.2 [63]). The demultiplexed sequences are deposited at the ENA under the accession number PRJEB81455.

### Fungal assessment by culturing

From 50 out of the 58 seed lots analyzed with Illumina MiSeq sequencing (Table 1), fungi were also assessed by culturing. As described in more detail in Franić et al. [6], a total of 100 seed halves per seed lot were incubated at room temperature on 1.5% water agar (PPA, Pronadisa Lab Conda, Madrid, Spain) including 100 mg/L streptomycin to remove bacterial contaminants. All fungi emerging from seeds within 21 days were transferred onto streptomycin-containing (100 mg/L) potato dextrose agar (Difco Bacto PDA, 39 g/L; ChemieBrunschwig AG, Basel, Switzerland). All isolates obtained from seeds belonging to the same tree genus were grouped together based on their macromorphology. Two to five representative isolates per morphotype (in total 441 isolates) were then identified by Sanger sequencing of their ITS region as described by Franić et al. [6] using the primers *ITS1* and *ITS4* [69] (Supplementary Table S1). For this study, the edited ITS sequences of the morphotypes published in Franić et al. [6] and deposited under GenBank accession numbers MN105153–MN105593 were used. The sequences were re-classified against the same UNITE database used for the metabarcoding datasets (Illumina and ONT) as described above.

### Fungal lifestyle assignment

Lifestyles (i.e., the functional traits) of the fungal OTUs obtained with the Illumina MiSeq metabarcoding and ONT metabarcoding were determined for fungi identified at genus level using the interactive lookup table FungalTraits, following the author’s instructions [70]. We note here that these functional assignments are based on the literature and reflect the most commonly occurring lifestyles for a given fungal taxon (i.e., genus) [70]. FungalTraits is an updated and extended version of FUNGuild [71], which was shown to perform better in assigning fungal taxa to plant pathogens than FUNGuild [72]. To discern plant endophytes (i.e., defined by Plõme et al. [70] as ‘asymptomatic, commensal or weakly mutualistic inhabitants’), plant pathogens and plant saprotrophs from other fungal lifestyle groups, we used the column ‘primary lifestyle’. Foliar and root endophytes were merged into the group ‘plant endophytes’, while litter, wood and nectar/tap saprotrophs were merged into the group ‘plant saprotrophs’. Entries of plant pathogens were defined as such.

### Data analysis

We first compared the overall number and taxonomic and lifestyle composition of fungal genera revealed by each of three metabarcoding datasets (Illumina MiSeq Taylor, Illumina MiSeq Tedersoo, ONT) and the culturing dataset. Afterwards, fungal alpha- and beta-diversity, considering OTUs, genus-level taxonomic and lifestyle assignments, were compared between metabarcoding approaches for different host tree groups and host tree species, with the latter representing a combination of host tree group and continent of origin, because seeds from single host tree species always originated from the same continent. The data analyses were conducted using *R* (version 4.2.0, [73]) in the programming environment *RStudio* [74]. To allow comparisons of composition and diversity between datasets, we focused on genus-level assignments across the Illumina, ONT and culturing datasets, as the lowest fungal taxonomic units in each method (i.e., OTUs, morphotypes) were generated separately and were thus not directly comparable.

#### Fungal community comparison between metabarcoding and culturing datasets

The total number of shared and unique fungal genera was compared between culturing and the metabarcoding datasets. Moreover, the taxonomic composition of seed fungi revealed by the different metabarcoding methods was visualized, also focusing on genus-level assignments. Fungal OTUs obtained from each method were grouped by genus, after which relative abundances for each fungal genus in each metabarcoding dataset were calculated. Relative abundances of the twelve most abundant fungal genera were then plotted across host tree groups (i.e., angiosperms and gymnosperms). To test whether certain genera were more likely to occur in either host group and if this was persistent across methods, we conducted enrichment analyses for each metabarcoding dataset separately. For this, Illumina datasets were rarefied (resampled) to 1,000 reads per sample and the ONT dataset to 164 reads. To determine if the employed sequencing depth sufficiently captured OTU and genus richness, rarefaction curves were calculated and visualized for the datasets (Supplementary Fig. S1). The rarefaction was done to account for differences in number of reads per sample within each dataset, and values were chosen to retain most of the samples (i.e., all samples in Illumina and around 62% of the samples in the ONT dataset). Quasi-Poisson generalized linear models (GLMs) were fit with the *R* function ‘glm’ to assess the differences in rarefied abundances of fungal genera (i.e., response variable) between angiosperms and gymnosperm (i.e., host tree group; explanatory variable) for each dataset separately. The use of GLMs for analyses of differential abundances in such way [75] is an alternative to the often-employed R-packages Deseq2 [76], and can be done using custom scripts, for example, see Mittelstrass et al*.* [77].

For a direct comparison of fungi on a sequence-base, the representative sequences of the Taylor, Tedersoo and ONT OTUs were matched against the ITS sequences and corresponding taxonomic assignments of the fungal cultures with BLAST. For this, the classification algorithm *classify-consensus-blast* [78] implemented in QIIME2 was used, with the *perc-identity* parameter set to 0.85, and the ‘maxaccepts’ parameter set to 5. This analysis was used to show what relative proportion of overall sequences in each metabarcoding dataset was represented by the cultures, without an interference of taxonomic assignment bias through the database or classification algorithm.

Besides describing fungal communities across samples based on their dominant fungal genera, this study investigated relative abundances of genera assigned to different fungal lifestyles (i.e., plant endophytes, saprotrophs and pathogens) between samples belonging to different host tree groups and sequencing methods. As described for fungal genera, to test whether certain lifestyles were enriched in either host group, Illumina datasets were rarefied to 1,000 reads per sample and 164 reads in the ONT dataset, and quasi-Poisson GLMs were used to estimate the prevalence of the fungal lifestyles for each host group.

#### Fungal alpha-diversity comparison between host tree groups and metabarcoding datasets

For the Illumina Taylor, Tedersoo and ONT metabarcoding datasets, alpha-diversity was measured as OTU and genus richness (i.e., the total number of OTUs or genera present in each sample). As richness is a metric that gives the same weight to abundant and rare taxa [79], it captures patterns of alpha-diversity of the whole fungal communities. In addition to this occurrence-based metric, alpha-diversity was estimated using abundance-weighted indices, i.e., the Shannon’s entropy and the Inverse Simpson Diversity [79, 80], allowing us to explore alpha-diversity patterns of dominant fungal communities. Abundance-weighted alpha-diversity metrices were calculated using the R package *hillR* [81], and only for Illumina data. In the case of the ONT dataset, abundance-based measures could not be calculated because all OTUs in the ONT dataset were singletons.

Effects of the metabarcoding method (i.e., Taylor, Tedersoo and ONT), host tree group (i.e., angiosperms and gymnosperms) and their interaction on fungal alpha-diversity (i.e., OTU and genus richness) were tested in generalized linear mixed models (GLMMs) using the ‘glmmTMB’ function from the R package *glmmTMB* [82]. We included host tree species nested in host tree group in the models as a random factor. We assumed a zero-truncated negative binomial distribution for the errors of OTU richness, which were non-zero counts, and a negative binomial distribution for the errors of genus richness which contained one sample with no identified genera. Although correcting for differences in the number of reads among samples by setting the offset argument to the log of the number of sequencing reads is recommended for alpha-diversity models [77, 83], in the main text we only present the results of the models without offset correction. Due to dataset-inherent differences in the number of reads per sample (i.e., lower numbers of reads per sample in ONT vs. Illumina), comparing alpha-diversity between normalized datasets can result in an overestimation of diversity in ONT vs. Illumina datasets. We present results of the models run with the offset parameter for read normalization in the Supplementary materials (Additional file 2). Significance of factors in each model was tested using the ‘Anova’ function from the *car* package [84]. A Tukey comparison of least-squares means was performed with the function ‘emmeans’ from the *emmeans* package [85]. The same function was used to calculate the estimated marginal means and standard errors.

To test how the proportion of genera assigned to the different lifestyles was influenced by host tree group and metabarcoding method, we used Chi-Square tests on binomial GLMs [86, 87], across the full sample set and using the formula ‘proportion ~ lifestyle + host group * method’ with the R function ‘glm’. Proportions in this case were used instead of absolute numbers to account for differences in genus richness between datasets.

#### Fungal beta-diversity comparison between host tree groups and metabarcoding datasets

To visually explore the fungal beta-diversity within each dataset, Principal Component Analysis (PCA) and hierarchical clustering were used. For both analyses, the read count table was normalized to centered-log ratio (CLR [88]), a method that corrects for the sparse nature of compositional data. For PCA, the Monte-Carlo instances were generated and obtained using the functions ‘aldex.clr’ and ‘getMonteCarloInstances’ from the R package *ALDEx2* [89]. The resulting normalized communities were then restricted to the 150 most abundant taxa in each sample for the analysis of beta-diversity at OTU level. As the 150 most abundant taxa partially varied between samples, this number added up to more than 150 taxa for each dataset. We also conducted a genus-level PCA, for which all genera identified in each dataset were used. The function ‘prcomp’ of the R *stats* package [73] was used to conduct PCA.

For community-level analyses using hierarchical clustering at genus level, each dataset was subset to the 50 most abundant genera across samples after CLR normalization. Heatmaps were then plotted with the R package *pheatmap* [90] using the Minkowski distance, a measure that describes the shortest distance between points in Euclidean space, as clustering method [91, 92].

The effects of ‘host tree group’ and ‘method’, and ‘host tree species’ and ‘method’, including interaction terms for both, on differences in fungal community structure (i.e., beta-diversity) at genus level were assessed with permutational multivariate analysis of variance (PERMANOVA) [93] using the ‘adonis’ function from the *vegan* package [94]. PERMANOVAs were run on the Aitchison dissimilarity matrix [88], which was calculated from merged raw abundance matrices containing OTUs assigned to genera across samples and datasets, using the ‘vegdist’ function from the *vegan* package with the method argument set to ‘robust.aitchinson’. Focusing on genus assignments allowed us to analyze the metabarcoding datasets at once, as merging datasets at OTU level would not have been possible due to the separately generated and not fully overlapping amplicons represented by those OTUs.

Additionally, to determine pairwise differences between samples belonging to different host tree groups but that were assessed with different methods, we used the ‘pairwise.adonis’ function with the method argument set to ‘fdr’ for *P*-value adjustments from the *pairwiseAdonis* R package [95], which is a wrapper for multilevel pairwise comparison of the ‘adonis’ function from the R package *vegan*. We focus only on pairwise comparisons at host tree group and method level because the number of comparisons for host tree species and method would result in 529 pairwise comparisons.

## Results

### Fungi revealed by the metabarcoding and culturing datasets

**Fig. 1:**
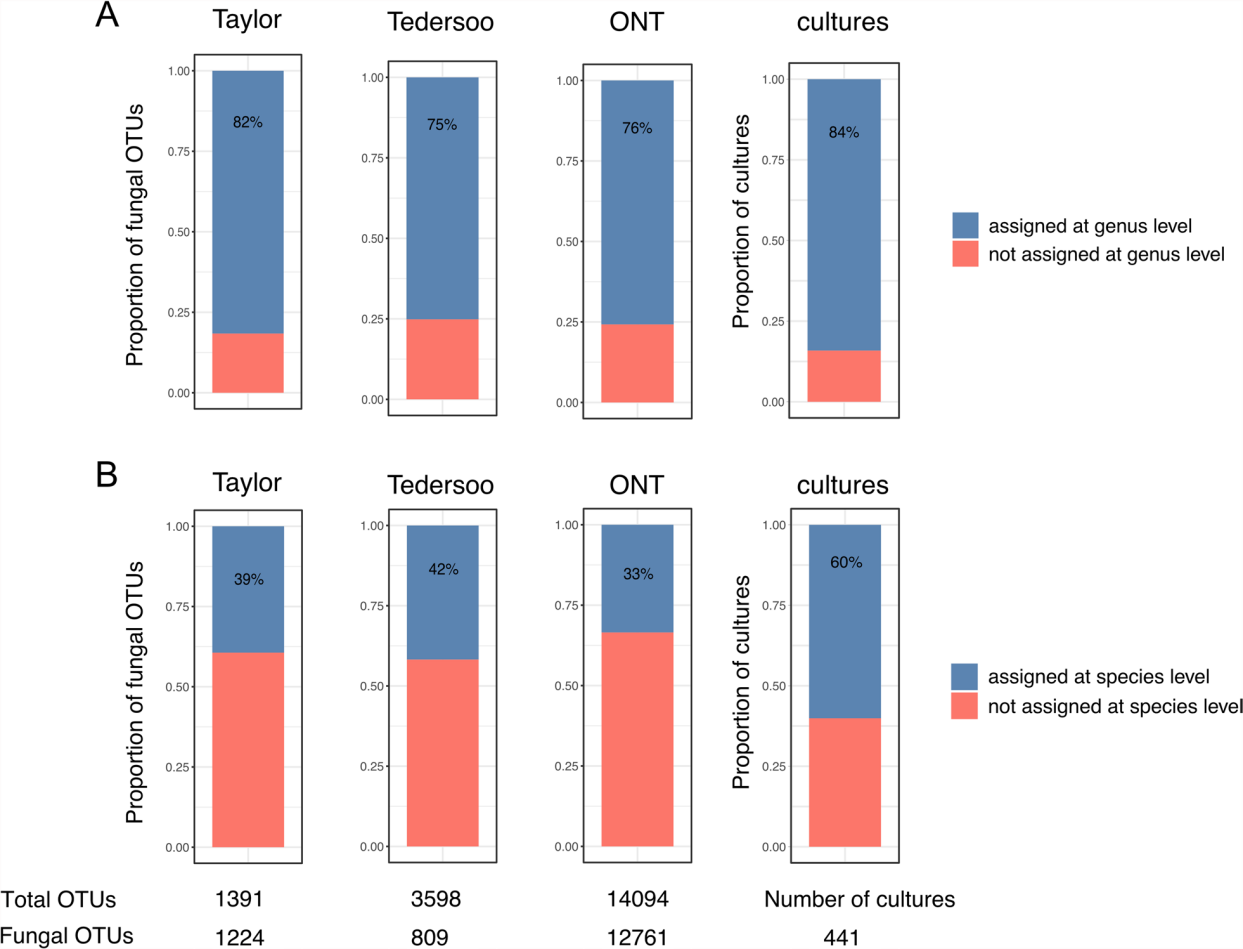
Proportions of fungal OTUs or cultures assigned to genera and species. The proportions of fungal OTUs from the Taylor, Tedersoo and ONT dataset or from the sequenced cultures that were assigned to **A** genera and **B** species. All sequences were matched to the same version of UNITE for taxonomic assignments. The total number of OTUs and number of fungal OTUs are indicated below the bars for each metabarcoding method, while the number of sequenced fungal cultures is indicated for the culturing dataset

After bioinformatic processing, Illumina MiSeq sequencing yielded a similar total number of reads for both datasets (Taylor: 2.49 M reads; Tedersoo: 2.48 M reads). Among those reads, which clustered into 1,391 and 3,598 OTUs, 11% and 66% could be assigned to plants in the Taylor and Tedersoo dataset, respectively. While the proportion of reads assigned to plants in the Taylor dataset was similar in angiosperms and gymnosperms, the Tedersoo dataset contained 44% of plant reads in angiosperm samples but 88% of plant reads in gymnosperm samples (Supplementary Fig. S2). A total of 1,224 (88%) and 809 (22.5%) fungal OTUs were found in the Taylor and Tedersoo dataset, respectively. From the fungal OTUs, around 82% were assigned to genera (39% to species) in the Taylor dataset and 75% to genera (42% to species) in the Tedersoo dataset (Fig. 1). Specifically, the numbers of fungal genera identified in the Taylor dataset was 244 (253 species) while 217 genera and 234 species were identified in the Tedersoo dataset.

The bioinformatic processing of the raw long-read ONT sequences resulted in 2.42 M reads. From these reads, 14,094 OTUs (all singletons) were obtained, of which 12,761 (90.5%) were fungal OTUs. From these fungal OTUs, 76% were assigned to 226 unique genera and 33% to 282 unique fungal species, respectively (Fig. 1).

The culturing approach identified a total of 441 fungal morphotypes based on morphological grouping of more than 4,000 isolates [6]. Based on Sanger sequencing of the ITS region, 371 morphotypes (84%) could be assigned to 62 unique fungal genera and 265 morphotypes (60%) to 72 unique fungal species (Fig. 1).

Similar results regarding the proportion of fungal OTUs assigned to genera and species were also observed when abundances (read counts) of the OTUs were considered. The relative abundances of reads belonging to fungal OTUs that were assigned to genera/species were similar across the datasets, with the Taylor dataset showing the highest proportions of fungal reads assigned to genera/species (84%/40%), and Tedersoo and ONT datasets following with similar values (79%/36% and 75%/37%, respectively).

In addition to summarizing fungal taxa across datasets, this study also investigated the distribution of fungi into different primary lifestyles (i.e., potential plant pathogens, plant saprotrophs or plant endophytes) based on their genus-level assignments. In the Taylor dataset, 39% of the fungal OTUs (480 out of 1,224) were assigned to one of the above-mentioned lifestyles, while around 46% of the fungal OTUs (373 out of 809) were assigned to a lifestyle in the Tedersoo dataset (Supplementary Table S4). Around 44% of fungal OTUs were assigned to a lifestyle in the ONT dataset (5,621 out of 12,761 OTUs), as well as in the culturing dataset (179 out of 441 morphotypes).

### Fungal community comparison between metabarcoding and culturing datasets

**Fig. 2:**
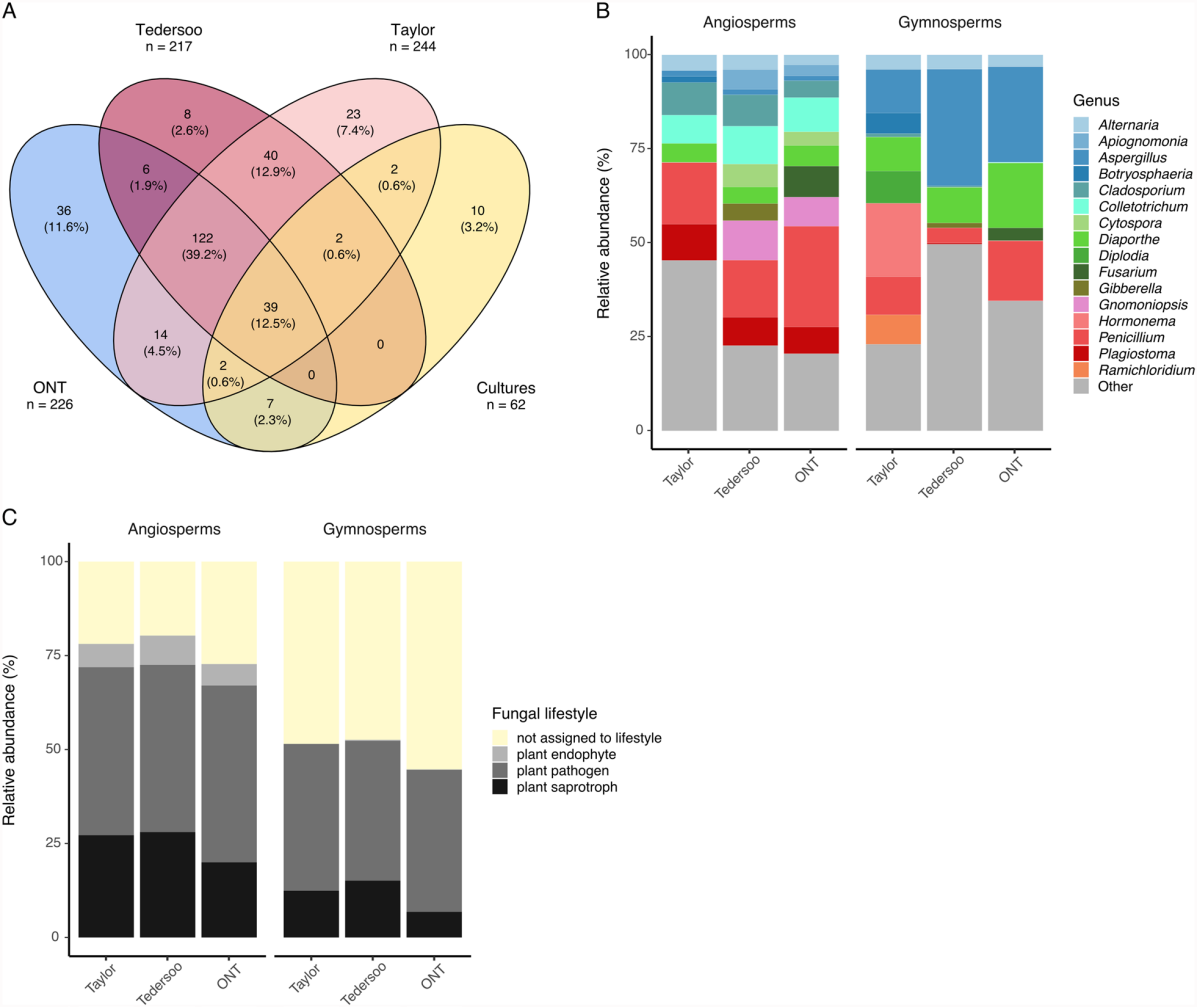
Differences in genus composition and lifestyles of the seed mycobiota across methods.** A** The overlap of unique genera identified across the Taylor, Tedersoo, ONT metabarcoding and culturing datasets. The numbers inside the Venn Diagram are genera counts and proportions related to the overall (total) number of genera identified across all methods (*n* = 311), while the total numbers of genera identified with each method are indicated outside of the diagram. **B** Community profiles of fungi identified in the seeds of the two host tree groups (angio-and gymnosperms), in the three metabarcoding datasets. A total of 26 angiosperm and 32 gymnosperm seed lots were analyzed for the Taylor and Tedersoo datasets, respectively, while 21 angiosperm and 27 gymnosperm seed lots were analyzed in the ONT dataset. The bar plot shows average relative abundances of taxa assigned at genus level, across samples within each host tree group. The twelve most abundant genera identified for each of the datasets were used to generate the plot. Thus, some genera with small average values in either host tree group may not be easily visible but are still present among the overall dominating genera within each dataset. Genera not among the top twelve taxa were summed up and labeled as ‘Other’. **C** The distribution of fungi identified at genus level into lifestyles and their average relative abundances in the seeds of the two host tree groups (angio- and gymnosperms) for the three metabarcoding datasets

A comparison of fungal genera identified from each of the four methods showed the largest overlaps across the three sequencing-based methods (Fig. 2A). Almost 40% of all identified genera (122 out of 311 overall identified genera, Fig. 2A) were found exclusively in the three sequencing-based datasets but not in the culturing dataset. The Taylor and Tedersoo datasets shared over 80% of their total genera, and each of them shared around 60% of the genera with the ONT dataset. Only 39 out of 311 genera (12.5% of all identified genera) were shared across all four datasets. Portions of dataset-specific genera in the metabarcoding datasets ranged from approximately 3% (8 out of 217 genera, Tedersoo dataset) to 12% (36 out of 226 genera, ONT dataset), and they belonged to all three investigated fungal lifestyles (Additional file 1). Noteworthy, ten genera (out of 62 cultured genera) were exclusively found by culturing (Additional file 1).

A direct comparison of sequences obtained from the three metabarcoding datasets against the sequences obtained by culturing revealed that more than 80% of the reads in the Taylor (88%) and Tedersoo (83%) dataset matched against the cultured fungi, while more than half of the reads of the ONT dataset could not be matched to sequences of the cultures but were assigned to species within the UNITE database (65%). The metabarcoding communities differed in their overlap with cultured sequences not only between the datasets, but also in dependency of the host tree group or even the sample (Supplementary Fig. S3).

Comparing the fungal communities between metabarcoding methods revealed that *Alternaria*, *Aspergillus*, *Colletotrichum, Diaporthe, Penicillium* and *Plagiostoma* were recorded among dominant genera in all three datasets (Fig. 2B). Some genera appeared in all datasets and both host tree groups in relatively high abundances, for example *Diaporthe*, which on average amounted to more than 4% in angiosperm seeds and more than 6% in gymnosperm seeds. At the same time, some of the genera dominant across datasets were differently abundant in angiosperms and gymnosperms, as indicated by the enrichment analysis (Supplementary Table S3). For example, the average relative abundance of *Colletotrichum* was higher in angiosperms than gymnosperms in all three datasets. *Plagiostoma*, *Cladosporium* and *Gnomoniopsis* were also more relatively abundant in angiosperms than gymnosperms (Fig. 2B), but this trend was significant only in the Taylor and Tedersoo dataset. *Penicillium* showed a similar trend in all three datasets, but differential abundances between host tree groups were significantly different only in the Tedersoo dataset. The opposite trend, i.e., higher relative abundances in the gymnosperm over angiosperm seeds, was observed for *Diplodia*, *Hormonema* and *Aspergillus* in all three datasets, but differences in *Aspergillus* were only significant in the Taylor and ONT, and in *Hormonema* only in the Taylor dataset (Supplementary Table S3). In dependency of host tree group and dataset, *Fusarium* was among the dominant genera only in the ONT dataset and enriched, although not significantly, in angio- vs. gymnosperms, while *Ramichloridium* was among the twelve dominant genera only in the Taylor dataset (Fig. 2B) and more frequent in gymnosperms than in angiosperms (Supplementary Table S3).

Of the genus-level assigned OTUs that were also assigned to a lifestyle, plant pathogens had the highest number of OTUs and OTU read abundances, followed by plant saprotrophs and endophytes (Supplementary Table S4). Relative abundances of the lifestyles were similar across datasets but varied to a certain extent between host tree groups (Fig. 2C). Endophytes overall were more prevalent in angiosperm seeds than in gymnosperm seeds (enrichment analysis: *P* < 0.05) in all datasets, although endophyte abundances varied among angiosperm samples. For example, unlike other angiosperm seeds, *Quercus garryana* seeds harbored endophytes in high relative abundances (> 30%) that primarily belonged to *Gnomoniopsis sp.* or *G. paraclavulata* in all datasets.

### Fungal alpha-diversity comparison between host tree groups and metabarcoding datasets

**Fig. 3:**
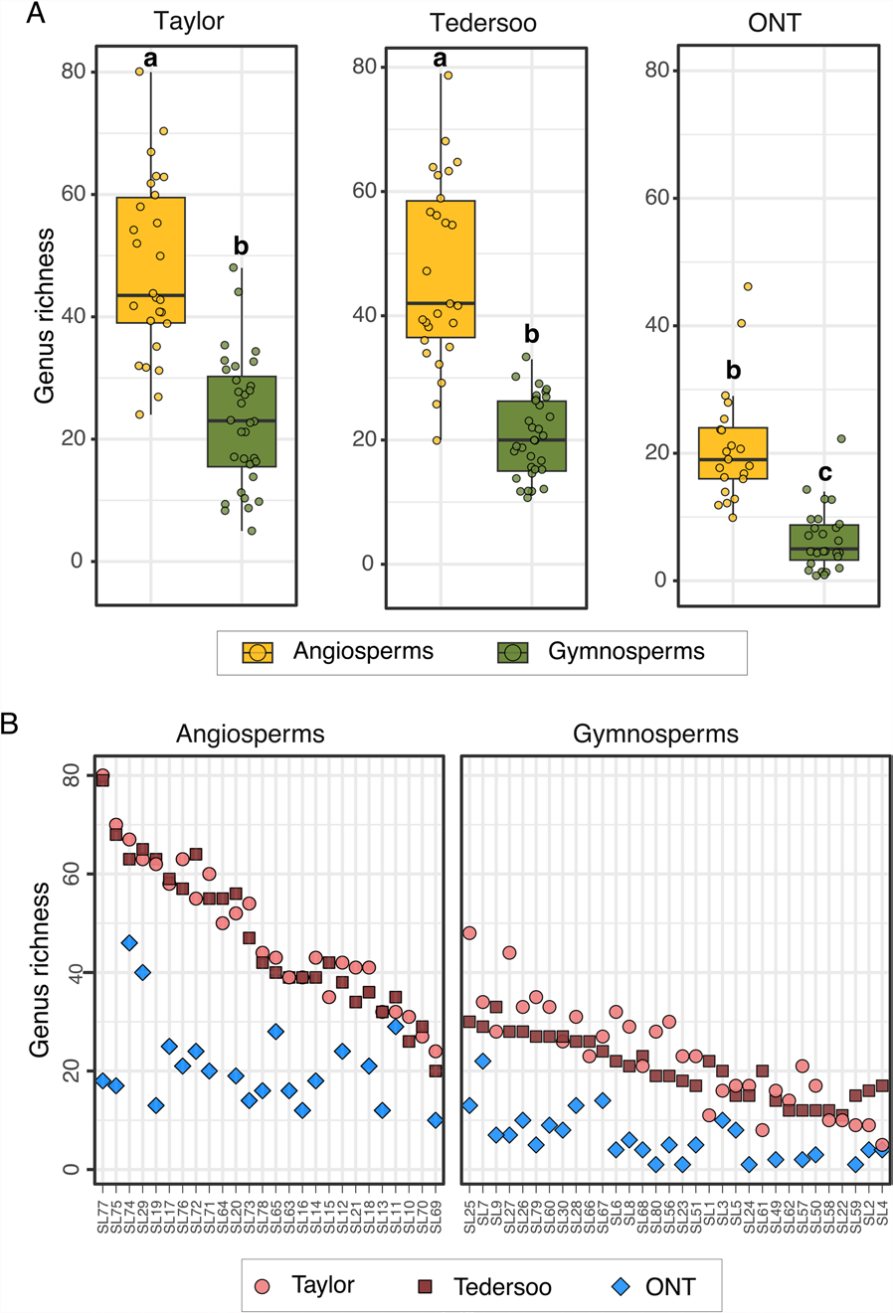
Alpha-diversity of seed mycobiota across host tree groups and methods.** A** Genus richness in the Taylor, Tedersoo and ONT datasets, in the angio- and gymnosperm seed fungal communities. Boxplots show the median and interquartile ranges for the plotted measures, and each point represents one sequenced sample. Different letters above the bars indicate significantly different values (*P* < 0.05). **B** Per-sample genus richness across samples belonging to different host tree groups and methods ordered based on the number of fungal genera per sample

A higher richness of fungal genera in angiosperm in comparison with gymnosperm seed samples (χ^2 ^= 39.01, df = 1, *P* < 0.001) was revealed (Fig. 3). However, genus richness was similar in the Taylor and Tedersoo datasets and higher than in ONT dataset (χ^2 ^= 97.00, df = 2, *P* < 0.001; Fig. 3A). Although the interaction term between host tree group and method was significant (χ^2 ^= 10.36, df = 2, *P* < 0.05), indicating a method-dependent trend of differences in genus richness between angiosperms and gymnosperms, this was not observed with post hoc testing (Fig. 3). Per-sample richness of genera was on average half as low in the ONT dataset compared to both Illumina metabarcoding datasets, that is, on average 47.5 (angiosperms) and 21.6 (gymnosperms) genera were identified per sample in the Taylor and Tederoo datasets, while 21.1 (angiosperms) and 6.5 genera (gymnosperms) were identified per sample by ONT sequencing. Fungal OTU richness was higher in the ONT than in the Taylor and Tedersoo datasets (χ^2 ^= 53.80, df = 2, *P* < 0.001), and in angiosperm than in gymnosperm samples (χ^2 ^= 24.68, df = 1, *P* < 0.001) in all three dataset (as indicated by a non-significant interaction between host tree group and method; χ^2 ^= 0.96, df = 2, *P* = 0.62; Supplementary Fig. S4A).

Based on abundance-weighted alpha-diversity measures (i.e., Shannon’s entropy and the Inverse Simpson Diversity) and the relative abundance of the most dominant OTU for the Taylor and Tedersoo datasets (Supplementary Fig. S4B-C), lower evenness was found in the gymnosperm than angiosperm communities in both datasets, indicating that the angiosperm seed communities consisted of a higher number of more evenly abundant OTUs than gymnosperms, which were dominated by a lower number of OTUs.

Considering fungal lifestyles, within each dataset, angiosperms overall hosted a slightly higher diversity of plant pathogenic genera and saprotrophic genera than gymnosperms (Supplementary Fig. S5). Plant endophytes were the least genus-diverse group in all three datasets and in both host groups. In addition, not all seed samples harbored genera with an endophytic lifestyle, and seed lots without plant endophytic genera were more often found in gymnosperms (Supplementary Fig. S5). A joint analysis across methods of the per-sample proportions of genera assigned to different lifestyles showed that the combination of method and host group significantly influenced the proportions of genera in the different lifestyles (*P* < 0.01, df = 8).

### Fungal beta-diversity comparison between host tree groups and metabarcoding datasets

**Fig. 4:**
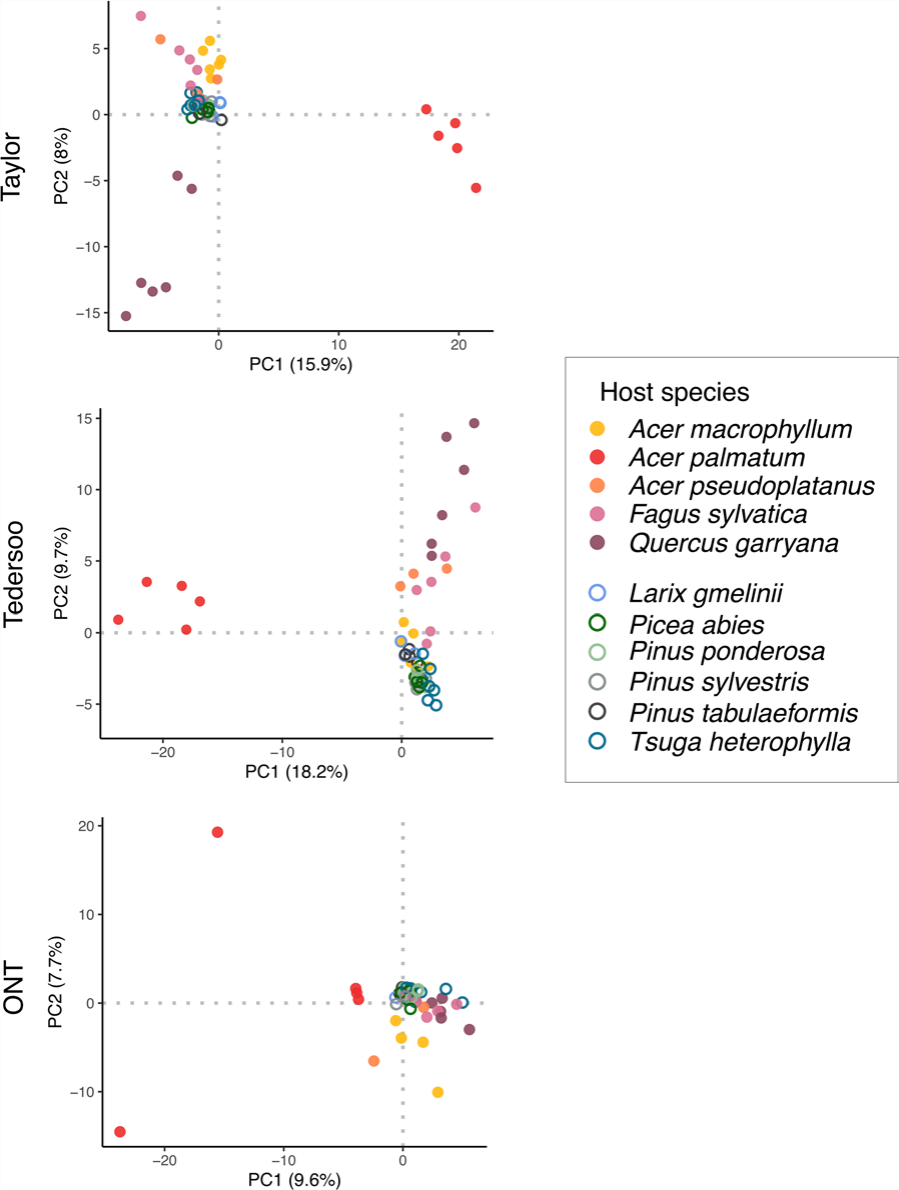
The structure of the mycobiota in seed samples from different host tree species. PCA plots are based on fungal genera revealed by the Taylor, Tedersoo and ONT dataset and their CLR-normalized abundances. The samples are colored according to host tree species, with filled points representing angiosperm and hollow points representing gymnosperm samples. The full set of genera identified within each dataset were used for the genus-level PCA analyses (Taylor: *n* = 244, Tedersoo: *n* = 217, ONT: *n* = 226)

PCA analyses were conducted to visually determine whether the fungal communities were structurally clustered based on host tree group and host tree species (indirectly related to their continent of origin). Fungal community structure at genus level in all three metabarcoding datasets revealed similar patterns related to host tree group and host tree species in Taylor and Tedersoo dataset (Fig. 4). For example, in addition to communities from the North American oak *Q. garryana*, communities from *Acer palmatum* repeatedly clustered apart from the main bulk of communities in the Taylor and Tedersoo dataset. In the ONT dataset, samples belonging to *A. palmatum* were also separated from the rest of the communities along PC1, but to a less striking extent. Overall, the proportion of variance explained by the first two PCs was higher in both Taylor (23.9%) and Tedersoo (27.9%) datasets in comparison with the less differentiated ONT data (17.3%). In all datasets, the gymnosperm seed samples did not spread along the axes as much as the angiosperm samples. Similar to fungal communities at genus level, the communities at OTU level in the Taylor and Tedersoo dataset showed separation based on a combination of host tree group and species (Supplementary Fig. S6). The communities of the Asian *A. palmatum* clearly differed from most of the other communities along PC2 in both datasets. The Taylor dataset further emphasized structural differences in communities of the North American *Acer macrophyllum*, while communities of the North American *Pinus ponderosa* separated from the bulk of communities in the Tedersoo dataset on PC1.

Similar results were obtained with PERMANOVA, which revealed that host tree group and method, as well as their interaction, had significant effects on the community composition of fungal genera (Table 2). However, only about 10% of variation in fungal communities across samples was explained by those factors, with host tree group alone accounting for 6% of the variation (Table 2). Structuring of the fungal communities was also observed at a host tree species level (Fig. 4)—the PERMANOVA model containing host tree species, method, and their interaction, in which all explanatory variables were significant (Table 2), explained almost 50% of variance with host tree species accounting for 33% alone. Furthermore, pairwise comparisons based on host tree group and method showed that the Taylor and Tedersoo datasets revealed similar fungal communities, which showed separation based on host tree group (angiosperms vs. gymnosperms; Supplementary Table S5). Differences between angio- and gymnosperm communities were also noticed in the ONT dataset, but those were different from the communities of angiosperm and gymnosperm trees in the Taylor and Tedersoo dataset (Supplementary Table S5).

**Table 2:** Results of PERMANOVAs testing for differences in community structure of the mycobiota of seeds

Factors	DF	F	R^2^	*P-*value
Method	2	1.610	**0.018**	**0.007**
Host group	1	10.925	**0.063**	**0.001**
Method * host group	2	1.742	**0.020**	**0.004**
Method	2	2.205	**0.018**	**0.001**
Host species	10	7.790	**0.326**	**0.001**
Method * host species	20	1.343	**0.112**	**0.001**

The results are based on genus-level community matrices for which the robust Aitchison distances were calculated. Degrees of freedom (DF), R^2^ and F values are shown. Significant *P*-values (*P* < 0.05) and corresponding R^2^ values are given in bold

These structural patterns were corroborated by community-level analyses of abundances in heatmaps, which highlighted clusters of genera that were consistently, that is, across all three metabarcoding datasets, differentially abundant in specific subsets of samples (Supplementary Fig. S7). For example, seven fungal genera formed a separate cluster of highly abundant genera in *A. palmatum* in both the Taylor and Tedersoo dataset (and *Pseudopithomyces* as additional genus in the Tedersoo dataset). Although five of those genera were also abundant in the ONT samples of *A. palmatum*, they did not form a separate cluster, and distinct genera were additionally identified as abundant. Both *Gnomoniopsis* and *Cytospora* were abundantly found in samples of North American *Q. garryana* tree seeds in all three datasets, as well as *Neocucrbitaria*, *Apiognomonia* and *Ramularia* in *Fagus sylvatica*. In certain host tree species, each dataset revealed further abundant genera: for *F. sylvatica*, *Phoma* was abundantly found in the Taylor and Tedersoo dataset, *Seimatosporium* in the Tedersoo and ONT dataset, and *Didymocyrtys* in the ONT dataset exclusively. Interestingly, community analyses revealed a higher level of homogeneity within the gymnosperm seeds in all three methods: samples from gymnosperm seeds generally clustered more closely together irrespective of host tree species or origin and shared more fungal genera, while angiosperm samples belonging to different host tree species or origins were distinct from each other (Fig. 3, Supplementary Figs. S6 and S7).

## Discussion

Despite the high diversity of fungal OTUs and genera revealed by metabarcoding approaches in our study, a substantial number of reads and OTUs could not be assigned to genera or species, and, contrary to our expectations, this applied to both short- and long-read metabarcoding. Third-generation sequencing technologies, to which ONT belongs, produce reads of up to hundreds of kilobases vastly exceeding the lengths that can be obtained by Illumina sequencing. Unlike other studies which found higher fractions of reads assigned to fungal species in ONT vs. Illumina metabarcoding [50], we could not confirm that more reads or OTUs were identified to species in our full-length ITS ONT vs. ITS2 Illumina datasets. One partial explanation for this result may be the general lack of fungal type strain reference sequences and a high estimated fraction of undescribed fungi [96]. Although the number of entries of full-length ITS sequences in reference databases is increasing [97], there is a need to continue generating sequences of previously and newly described species. On one hand, this can be accomplished by intensifying and optimizing culturing and taxonomic classification to improve taxonomy-based assignments. On the other hand, sequence-based phylogenetic placement of fungi may become an important alternative for overall fungal identification [51, 54]. These two approaches should not be mutually exclusive. Another partial cause for the limited success in OTU assignments to genera and species in our ONT dataset may be related to the high total error rates (approx. 7.5%) characteristic of the ONT version (R9.4.1) [66, 67] that was used in our study. Recently, ONT error rates have been significantly reduced [47, 98] which, together with the continuous expansion of reference databases [26] and a better understanding of ONT sequence quality [99] to develop streamlined processing options, will likely result in higher taxonomic resolution of long ONT reads.

### Primer selection

Although there was a substantial overlap of the fungal genera recovered by short- and long-read metabarcoding, shifts in the ranking of genera according to their relative abundance were observed between methods and hosts. For example, the genus *Fusarium* was identified among the top twelve genera in the ONT dataset, while it was not among the top genera in the two Illumina metabarcoding datasets. Such discrepancies may be due to the preferential amplification of certain taxa by different primers depending on the part of the ITS region sequenced [39, 42, 100]. Therefore, metabarcoding primers should be optimized to ensure the detection of the target taxa [36, 53, 57, 101, 102], and ideally, multiple primer pairs should be used to ensure the amplification of the broader fungal community.

Primer selection is critical not only because different primer pairs may target different marker genes, resulting in amplification of distinct fungal community profiles [103], but also because primers may affect the depth and thus resolution of a metabarcoding assay due to co-amplification of host (plant) DNA. In this study, the *ITS3ngs-mix/ITS4ngsUni* primer pair used in the Tedersoo dataset generated 2.49 million sequences, of which 1.63 million (65%) were plant reads. The use of non-specific primers and co-amplification of plant reads in metabarcoding studies may reduce the probability of capturing all fungi in sequenced samples, especially those belonging to rare fungal taxa. However, our results show that the Tedersoo data consisted of similar fungal genera as the Taylor dataset. This implies that the sequencing depth was sufficient to capture most of the fungal diversity despite a large fraction of reads being ‘wasted’ on plant sequences, which was especially the case for certain gymnosperm samples. Thus, reducing plant co-amplification by selecting primers specific for fungi or using peptide nucleotide blockers to block host contamination when using non-specific primers [104, 105], can lead to a more complete assessment of fungal diversity.

### Fungal culturing

Culture-free (i.e., metabarcoding) approaches revealed more than three times the number of fungal genera compared to the culture-based approach. Greater richness in metabarcoding than culturing datasets has been repeatedly observed in previous studies of plant mycobiomes [6, 106, 107] and likewise in oomycete microbiomes [108]. However, in our study most of the dominant taxa from the Illumina datasets were also cultured, as it was previously observed for foliar fungi of *F. sylvatica* [109], suggesting that higher observed diversities in metabarcoding datasets partially stem from rare taxa. A unique feature of the culture-based approach is that only living and thus metabolically active microbes, except obligate biotrophs, can be cultured. Hence, a smaller number of taxa can be expected to be identified with this method compared to metabarcoding, which also detects relic DNA [110], DNA belonging to metabolically inactive organisms, and to obligate biotrophs. Furthermore, in our study, several fungal taxa were observed uniquely in the culturing dataset, similar as previously described by Oita et al. [106], which suggests that culturing and metabarcoding should be used simultaneously if possible. Since pure cultures are indispensable for follow-up experiments related to fungal trait assessment or interactions with host plants or other organisms, additional effort in this area could improve culture-dependent diversity assessments. Some of the possibilities include cutting plant tissues into small pieces or even grinding them before placing them on agar media to reduce competition among fungi and to allow rare and slow-growing fungi to grow [23, 111]. In addition, different growing conditions and multiple nutrient media, including those supplemented with plant material, can be used to ensure that a wide range of endophytes can be captured [21].

### Comparison of metabarcoding datasets

The ONT dataset revealed lower genus richness and a different fungal community structure than the Taylor and Tedersoo datasets. As previously described by Furneaux et al. [51], the higher frequency of rare taxa due to greater per-sample sequencing depth in short-read sequencing compared to long-read sequencing can contribute to lower per-sample genus diversity in long-read datasets, and this was likely the case in our study. A large amount of reads originally generated by the ONT sequencing was discarded with the bioinformatic pipeline, in addition to erroneous sequences and disregarding other initially sequenced markers (SSU and LSU). These processing steps, together with the high error rates which were discussed before, likely contributed to the reduced number of genera identified per sample. These limitations should be considered when samples with high expected diversity (including rare taxa) are studied, which may have applied to the angiosperm samples of our study. In our case, the discrepancy of observed genus richness between the Illumina and ONT datasets was more prominent for the angiosperm samples than for the gymnosperm samples, which was likely due to differences in observed proportions of rare OTUs in these two groups of samples and their relative contribution to fungal diversity. Interestingly, a study using ONT and Illumina to characterize fungi associated with tall fescue [50] found a more diverse community and more rare taxa in the ONT dataset, stressing that no absolute agreements exist regarding the outcome of using ONT across studies. Loss of rare taxa may also affect beta-diversity patterns – in our study, short-read metabarcoding revealed more structured communities along host tree species than long-read metabarcoding. This result aligns with our expectation that ONT, given its lower sequencing depth and higher error rates, will have reduced diversity coverage power compared to Illumina sequencing. ONT sequencing with newer MinION flow cells can be used to detect specific pathogenic fungi [47], which has proven useful in the field of diagnostics, where rapid approaches and long reads are needed [112, 113]. For diagnostic purposes, it should however be clear that important considerations about sensitivity (detection limits) and quantitative reliability would have to be made [55].

### Ecological patterns across metabarcoding datasets

Despite the differences in genus richness and fungal community composition between short- and long-read metabarcoding, higher richness of fungal genera in angiosperm seeds compared to gymnosperm seeds was observed across datasets. The trend of higher alpha-diversity in angiosperms than gymnosperms was previously observed in a global study of tree seeds from botanical gardens [4]. Given that sexual reproduction, seed development and seed structures show remarkable discrepancies between angiosperms and gymnosperms [114], the variation in fungal diversity as a function of host plant group is not surprising. This study also revealed differences in fungal community composition between host tree groups and host tree species, and these host-dependent patterns were visible in all three datasets. While the gymnosperm samples clustered more closely together in the PCAs and did not create distinct clusters in the heatmaps, the fungal communities among the angiosperm samples showed the opposite tendencies, indicating that tree species-dependent community structure was stronger in angiosperms than in gymnosperms. Phylogenetic proximity of the hosts and host functional traits were previously shown to influence microbial composition of fungal endophytes in tropical seeds [115], tree twigs [116], leaves [107, 117], roots [118] and bark [119], suggesting co-evolution of plants and their associated mycobiomes. The breadth of angiosperm clades and the evolutionary success of flowering plants [120] may thus have contributed to the concurrent explosion of fungi associated with them, while the phylogenetically older and less divergent gymnosperm tree species may instead host a more similar mycobiota.

Besides comparing the fungal taxonomic diversity revealed by different methods, our study also examined potential fungal lifestyles (i.e., plant pathogens, plant saprotrophs and plant endophytes) captured by different methods. Currently, little is known about the function of most seed-borne fungi, but primary lifestyle assignments can be made based on genus-level taxonomy using tools such as FUNGuild [71] or FungalTraits [70], as functional traits are often conserved at the genus or sometimes even higher taxonomic level. However, a large proportion of OTUs in metabarcoding studies cannot be assigned to a genus and therefore to a lifestyle, or lifestyle information is simply missing. This problem is amplified by the previously highlighted structure of reference databases commonly used for taxonomic assignment, with entries traditionally stemming from cultured type strains. Unculturable taxa or taxa without reference sequences, at the moment, cannot be taxonomically placed (i.e., so-called ‘dark’ taxa [121]) and thus cannot be used to further decrypt potential function. For example, in our study, approximately 50% of fungal OTUs were assigned to one of the three targeted lifestyles in all metabarcoding datasets, similar to a previous study in which half of the fungal OTUs obtained from leaves and needles of several tree species were assigned to a lifestyle [122].

Of the fungal OTUs assigned to a lifestyle, plant endophytes were less abundant in the seed samples than plant saprotrophs and plant pathogens, respectively, and this was observed across datasets. In our study, around 3% of the genera identified in each dataset were assigned to plant endophytes. Partly, this pattern may be explained by a low number of endophyte records in the FungalTraits database [70], i.e., only around 1% of more than 10,000 entries are assigned to plant endophytes. One reason for the lack of fungal endophytes in the database could also be due to their biotrophic nature, making them difficult to isolate, describe and name [123], and to a historical research bias towards plant pathogens. Finally, fungal lifestyles vary from pathogenic to mutualistic depending on numerous abiotic and biotic factors that may change over time [124]. Thus, the taxonomy-based lifestyle assignments of our samples represent only a snapshot of all potential lifestyles the mycobiota can have. To better understand fungi-host interactions, more research is needed. This could include metagenomic or metatranscriptomic experimental studies to assess the functional potential or active genes of entire mycobiome communities [112], for which new long-read sequencing technologies can be a useful approach.

## Conclusions

Here, we used metabarcoding (short-read and long-read sequencing) and culturing to assess fungi from the same seed samples belonging to several tree species. The datasets were compared to understand if similar fungal communities were recovered by all employed methods. Across the three metabarcoding datasets, similar absolute numbers of fungal genera and species were revealed. About half of the characterized fungi overlapped, but each dataset also identified unique taxa, depending on the host tree group and species. The discrepancies in taxonomic composition between metabarcoding methods may be indirectly related to processing steps (e.g., primer selection and bioinformatic processing) and sequence length, but also the relatively higher error rate in the ONT dataset due to old chemistry. This may have also influenced the higher relative fraction of reads in the Illumina datasets that were assigned to cultures compared with the ONT dataset. Despite these differences, similar host-dependent alpha- and beta-diversity patterns and functional lifestyle compositions were revealed. For biodiversity studies, Illumina metabarcoding might be a suitable method to use thanks to its high diversity coverage, affordable price and availability of established processing pipelines. Along with ongoing reductions of error rates and the population of reference sequence databases, we see opportunities of long-read ONT sequencing as a tool for diagnostic purposes, where the user is interested in confirming known species. Higher accuracies and sufficient sequencing depths provided, ONT is also advancing in community analysis. Finally, for research aiming at assessing fungal traits, the sequencing and archiving of pure cultures are still indispensable.

## Supplementary Information

Additional file 1.

Additional file 2.

Additional file 3.

## Data Availability

Sequencing data from this study is deposited at ENA under accession numbers PRJEB81281 (Tedersoo) and PRJEB81455 (ONT), and data previously generated by Franić et al. (2019) under PRJNA550270 (Taylor) and MN105153–MN105593 (cultured sequences). The scripts required for data analysis and plotting, and the metadata are deposited at bitbucket (https://bitbucket.org/janami/tree_seed_metabarcoding_methods).
