# Supplementary material for: Metabarcoding with Illumina and Oxford Nanopore Technologies provides complementary insights into tree seed mycobiota
Source: Environ Microbiome. 2025 May 19;20:53. doi: 10.1186/s40793-025-00712-7 (PMC12090628; doi:10.1186/s40793-025-00712-7)
Supplement: Supplementary file 3 — Additional file 3. [file 40793_2025_712_MOESM3_ESM.docx]

# Metabarcoding with Illumina and Oxford Nanopore Technologies Provides Complementary Insights into Tree Seed Mycobiota

**Supplementary Information**

Jana Mittelstrass^1*^, Renate Heinzelmann^1^, René Eschen^2^, Martin Hartmann^3^, Quirin Kupper^1^, Salome Schneider^1^, Simone Prospero^1^, Iva Franić^1^

Author Affiliations:

^1^ Eidgenössische Forschungsanstalt für Wald, Schnee und Landschaft WSL, Zürcherstrasse 111, 8903 Birmensdorf, Switzerland

^2^ CABI, Delémont, Switzerland

^3^ Institute of Agricultural Sciences, ETH Zürich, Zürich, Switzerland

## Supplementary Figures

**
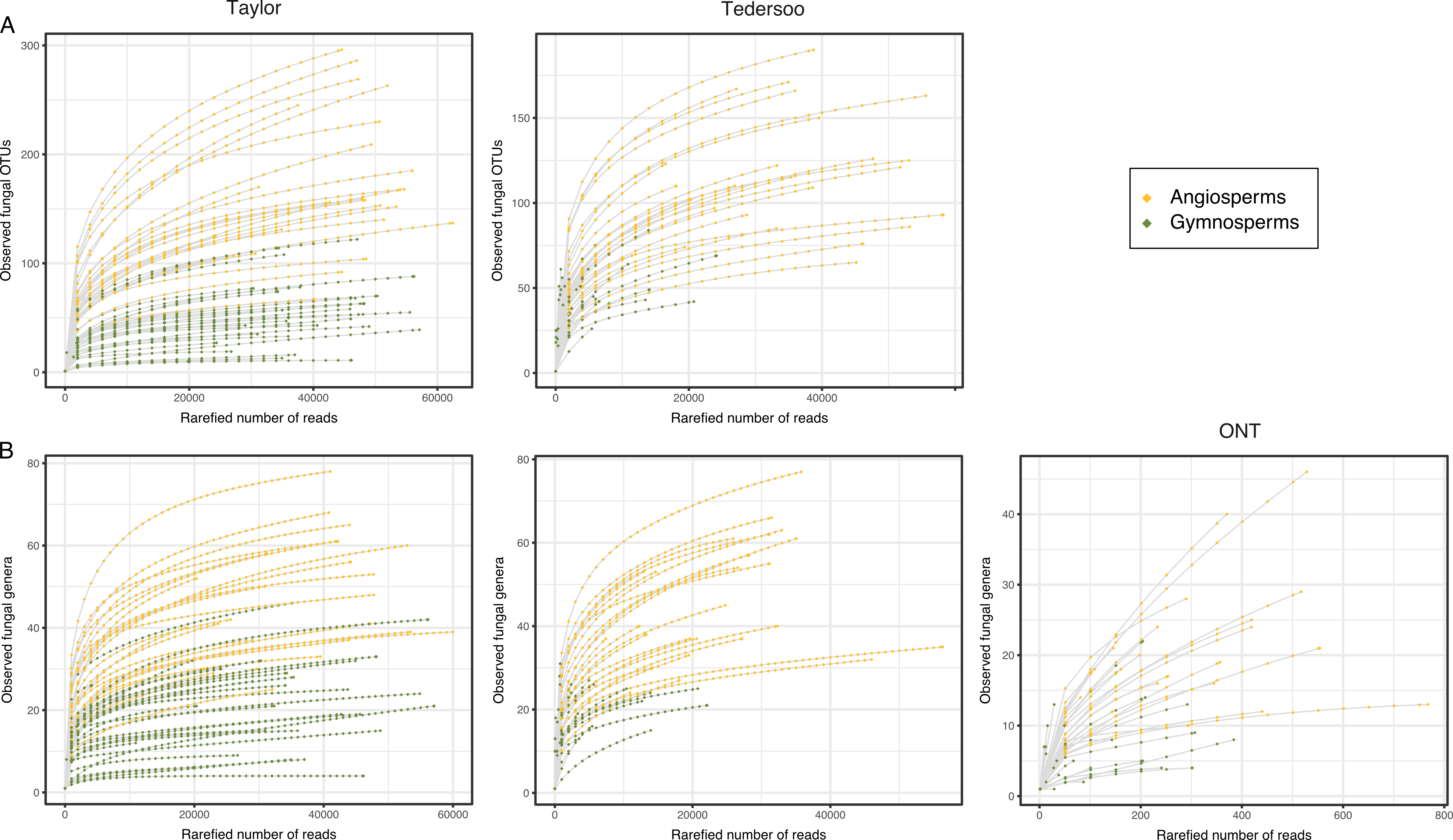
**

**Supplementary Figure S1.** Rarefaction curves for fungal OTUs (A) and fungal genera (B) were used to assess if the employed sequencing depth captures diversity across samples, for the Taylor (left), Tedersoo (middle) and ONT (right) metabarcoding datasets. Rarefaction curves at OTU level were not calculated for the ONT dataset due to its structure.


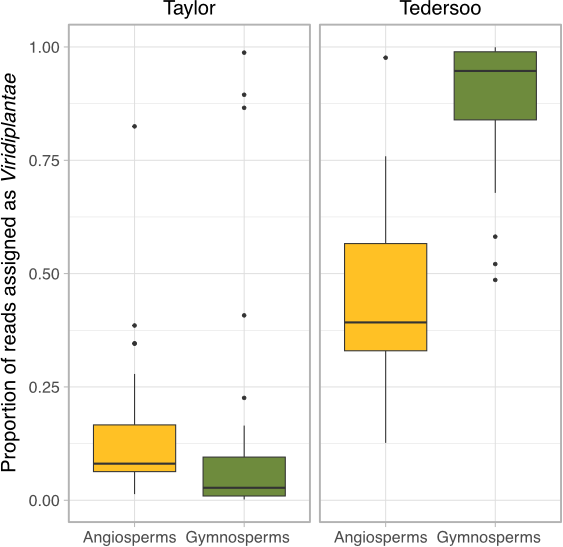


**Supplementary Figure S2.** The relative proportion of reads assigned to the kingdom *Viridiplantae* in the full UNITE database, in the Taylor (left panel) and Tedersoo (right panel) Illumina MiSeq datasets. Boxplots show the median and interquartile ranges for the plotted measures, and points represent sequenced samples. Yellow boxplots correspond to angiosperm samples and green boxplots show gymnosperm samples.


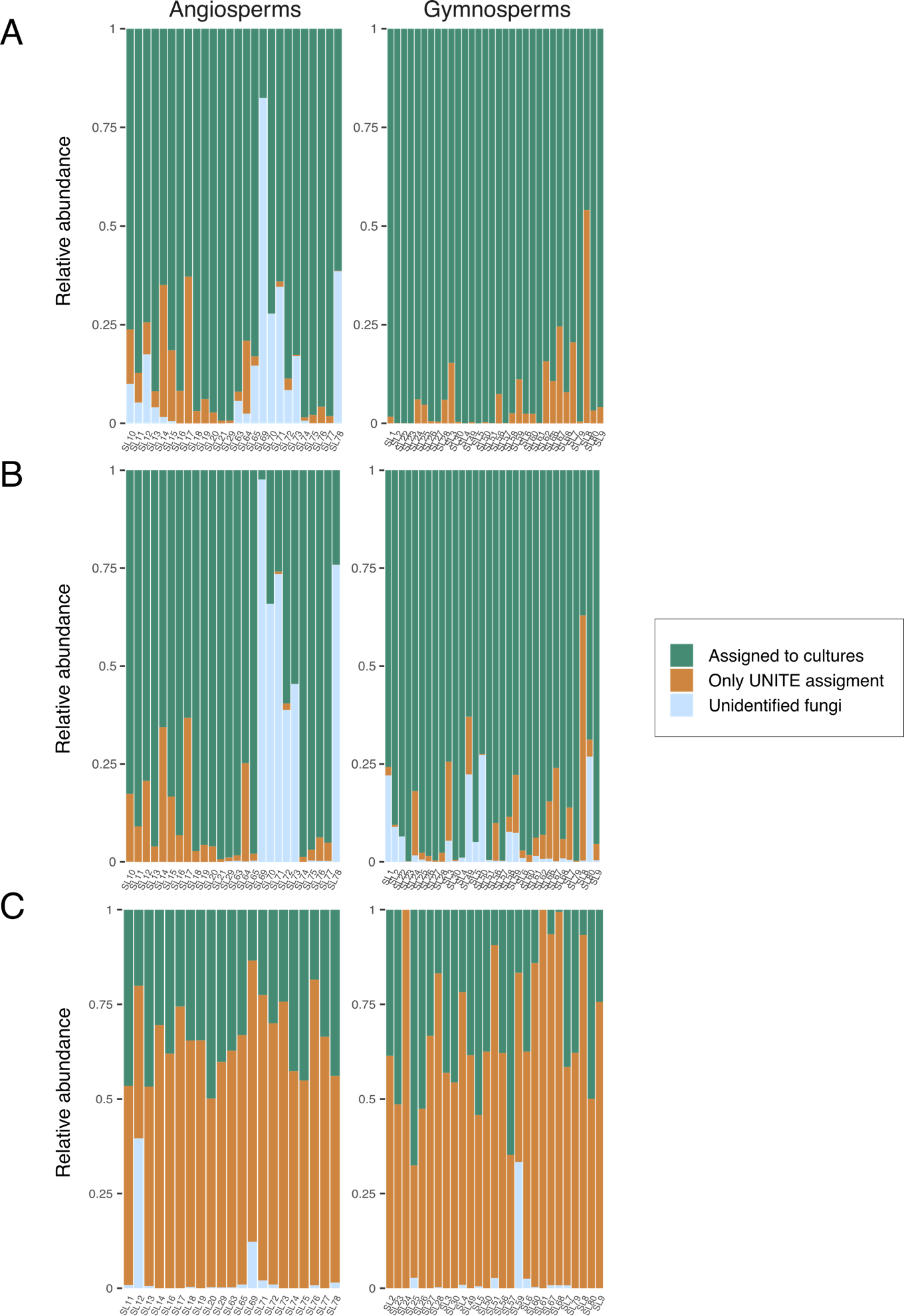


**Supplementary Figure S3.** Proportions of per-sample fungal metabarcoding reads matched to sequences of the cultured morphotypes, in the (A) Taylor, (B) Tedersoo, and (C) ONT dataset. For each sample, relative abundances were calculated for reads matching to sequences of the culturing dataset (green), reads not matching the culturing dataset but assigned to other fungi in the UNITE database (brown), and reads not assigned to the culturing dataset nor to classified entries within UNITE (blue). The samples (seed lots) are separated based on host tree group (i.e., angio- and gymnosperms). The relative abundances are based on all fungal reads of the sequenced community including singleton reads, to be consistent between the ONT and the Illumina metabarcoding datasets.


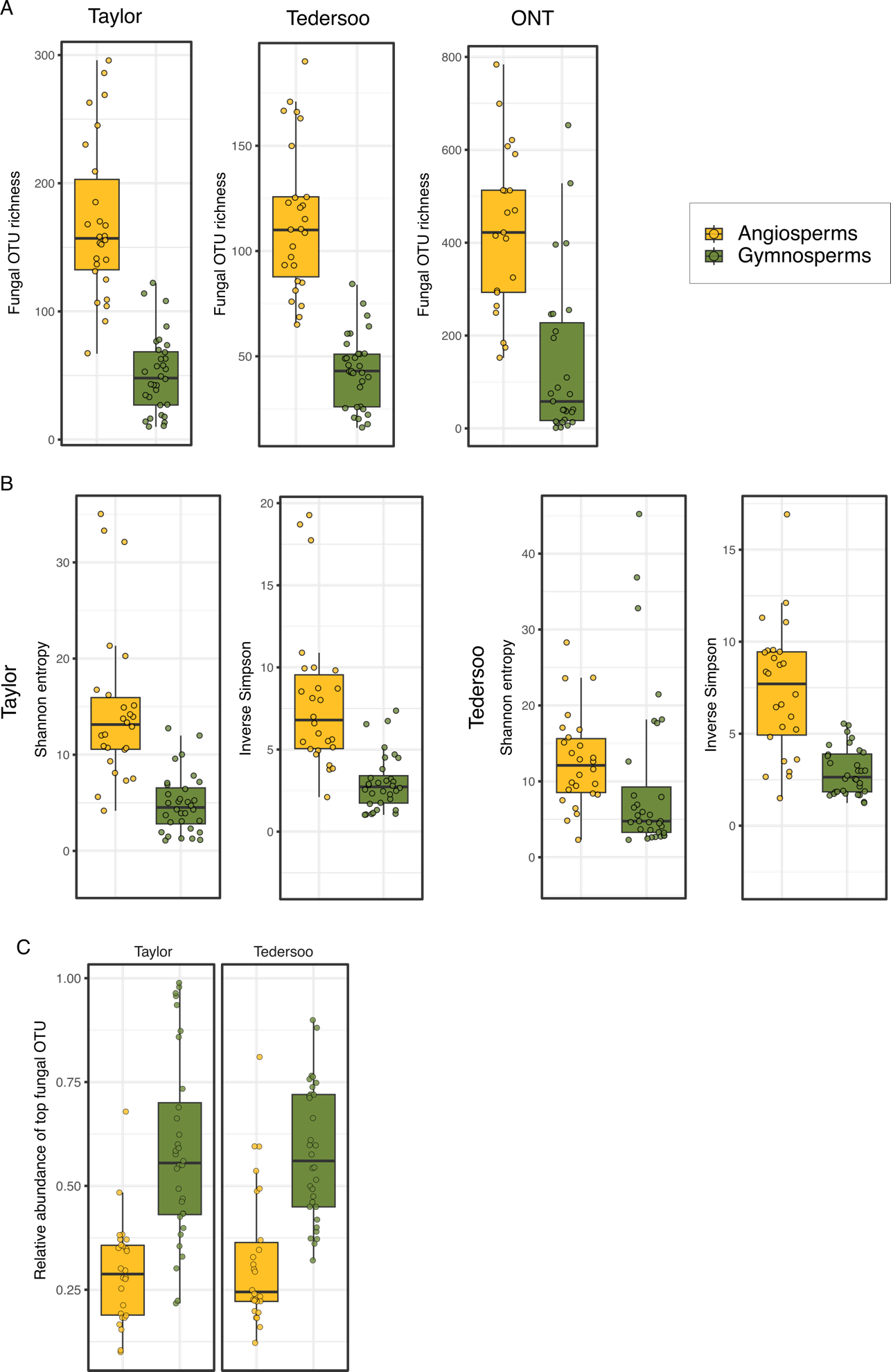


**Supplementary Figure S4.** Alpha-diversity measures based on fungal communities at OTU level in the seeds of angiosperms and gymnosperms. (A) Fungal OTU richness in the Taylor, Tedersoo and ONT dataset. (B) Shannon’s entropy and the Inverse Simpson Diversity in the Taylor (two left panels) and in the Tedersoo (two right panels). (C) Box plots of the relative abundances of the most abundant fungal OTU in each sample; an estimate for dominance. All boxplots show the median and interquartile ranges for the plotted measures, and each point represents one sequenced sample. We provide a short explanation of plotted indices to ease interpretation: OTU richness = number of taxa (in this case, OTUs) found in each sample, is higher in more diverse samples; Shannon entropy = uncertainty measure indicating the abundance-weighted proportions of discovered taxa, is higher in more diverse samples; Inverse Simpson = measure of evenness, is higher in more even and diverse samples with lower dominance; Relative abundance of dominant OTU = proxy of evenness (equitability) as it shows the extent of dominance, is high for samples dominated by single taxa (OTUs) (Chao et al., 2013; Jost, 2006; Oksanen, 2022).


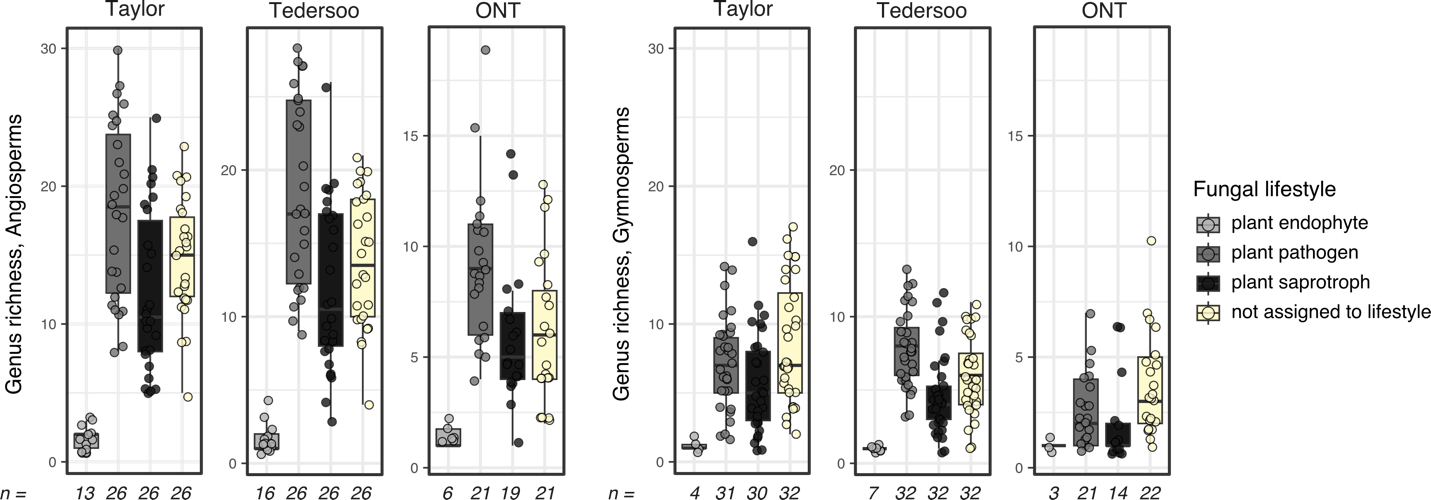


**Supplementary Figure S5.** Richness of genera within different fungal lifestyles as determined with FungalTraits. For both analyses and plots, only OTUs that were assigned to genera were considered: 999/1394 (Taylor), 608/3598 (Tedersoo), 9668/14’094 (ONT). All boxplots show the median and interquartile ranges for the plotted measures, and each point represents one sequenced sample. The sum of samples that hosted fungi in each lifestyle group within each datasets are displayed below the corresponding boxplot, in comparison to the total number of sequenced seed samples (26 angiosperm samples in the Illumina and 21 angiosperm samples in ONT, and 32 gymnosperm samples in the Illumina and 27 gymnosperm samples in ONT sequencing).

**
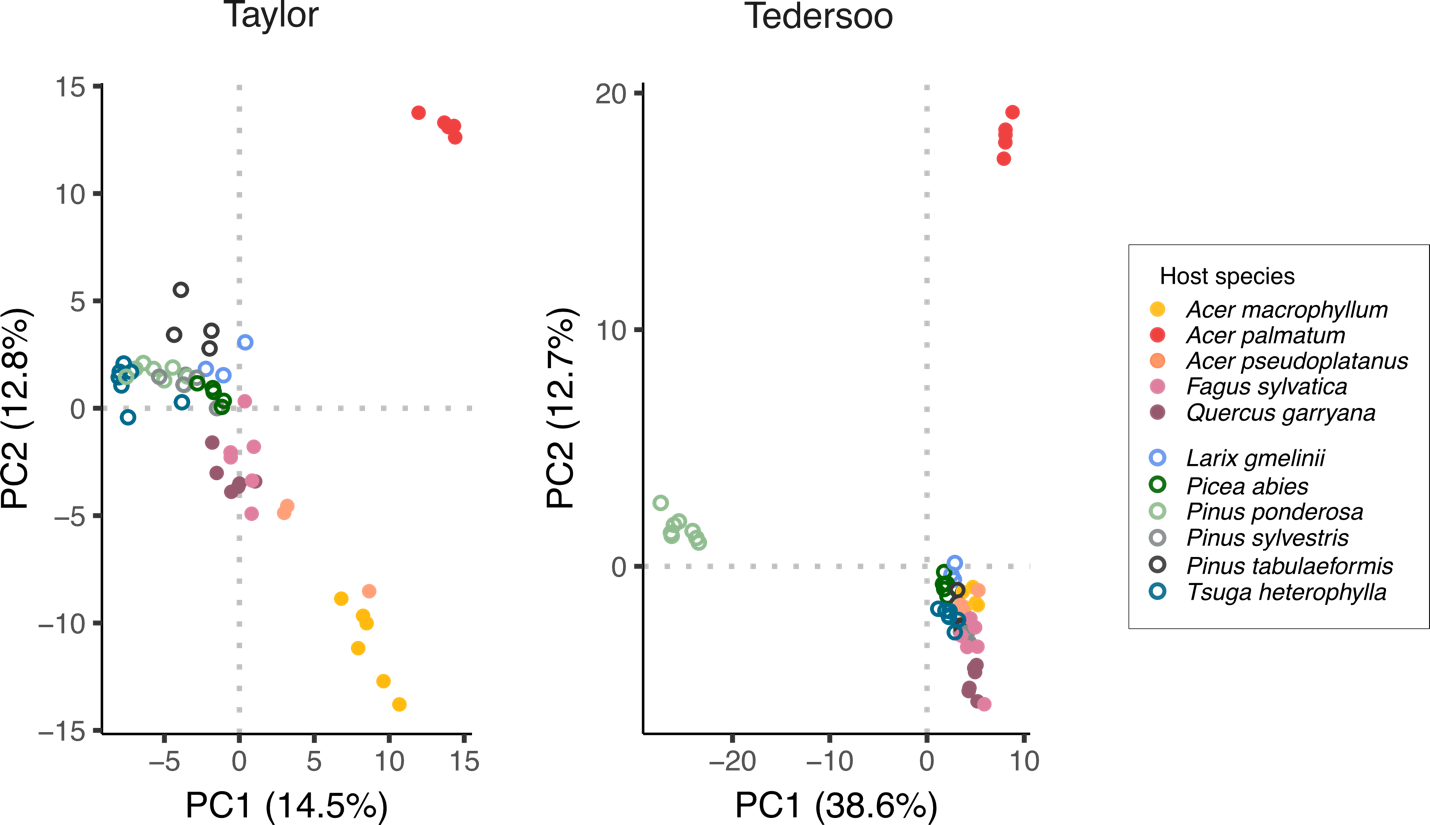
**

**Supplementary Figure S6.** The structure of the mycobiome characterized by the Taylor and Tedersoo metabarcoding datasets at the OTU level. The PCA plots show coloring based on host tree species. The top-most abundant 150 OTUs after CLR normalization were used for conducting PCA.


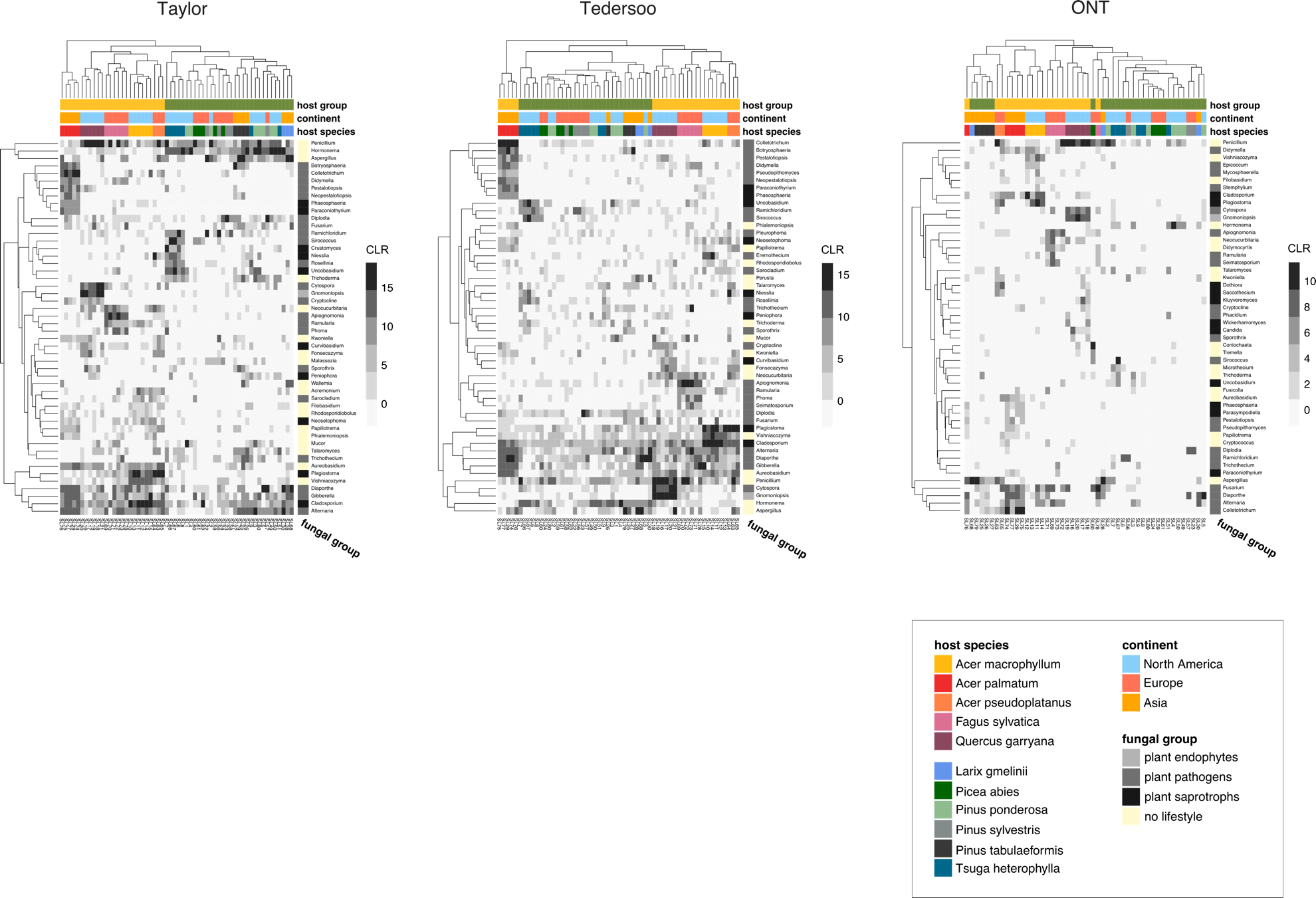


**Supplementary Figure S7.** Abundances of the 50 top genera in the seed mycobiota from the Taylor, Tedersoo and ONT datasets (left to right). The communities were first subset to the taxa that were identified to genus level and their abundances were normalized to CLR before restricting to the 50 most abundant genera in each dataset. The clustering was based on the Minkowski distance, and the genera (in rows) are colored according to membership of their primary lifestyle group. Samples are plotted in columns and the continent of origin, host tree group and host tree species are indicated above.

## Supplementary Tables

**Supplementary Table S1.** Primers used in the Illumina MiSeq and ONT metabarcoding, and primers used for the sequencing of the fungal cultures.

| **Library name, Fragment length, Region** | **Forward primer name and sequence** | **Reverse primer name and sequence** |
| --- | --- | --- |
| Taylor  (Franić et al. (2019))  ~ 330-340 bp  ITS2 | *5.8S-Fun* ^1)*^  AACTTTYRRCAAYGGATCWCT | *ITS4-Fun* ^1)*^  AGCCTCCGCTTATTGATATGCTTAART |
| Tedersoo  ~ 380-390 bp  ITS2 | *ITS3ngs-mix*^2)*^  CANCGATGAAGAACGYRG | *ITS4ngsUni* ^3)*^  CCTSCSCTTANTDATATGC |
| ONT  ~ 4-6 kB  Ribosomal operon *** | *NS1short* ^4)**^  CAGTAGTCATATGCTTGTC | *RCA95m* ^4)**^  CTATGTTTTAATTAGACAGTCAG |
| Culturing  ~ 530 bp  ITS1-ITS2 | *ITS1* ^5)^  TCCGTAGGTGAACCTGCGG | *ITS4* ^5)^  TCCTCCGCTTATTGATATGC |

All sequences in direction 5’ - 3’.

Original Publications: ^1)^ Taylor et al. 2016, ^2)^ Tedersoo et al. 2014 and Tedersoo 2015, ^3)^ Tedersoo and Lindahl 2016, ^4)^ Wurzbacher et al. 2018, ^5)^ White 1990

*The primers used for the amplification of both the Taylor and Tedersoo library in the first PCR included the Illumina flow cell adapters CS1 ACACTGACGACATGGTTCTACA[fwd] and CS2 TACGGTAGCAGAGACTTGGTCT[rev].

**The primers used in the ONT library construction were tailed on the 5’ end with the following adapters: TTTCTGTTGGTGCTGATATTGC [fwd], ACTTGCCTGTCGCTCTATCTTC [rev] as required by the ONT PCR barcoding Kit EXP-PBC096.

***During bioinformatic processing, the full-length ITS region was extracted from the initial ONT amplicon that spanned the SSU, the ITS region, and part of the LSU.

**Supplementary Table S2.** Overview of samples sequenced with the ONT metabarcoding approach and barcodes used to multiplex samples on runs. The raw reads column represents the number of reads for each sample after demultiplexing.

| **Sample**  **ID** | **Run**  **ID** | **Barcode ID** | **Barcode sequence** | **Raw reads** | **Flowcell ID** |
| --- | --- | --- | --- | --- | --- |
| SL25 | run1 | BC07 | GTGTTACCGTGGGAATGAATCCTT | 39506 | FAK47171 |
| SL75 | run1 | BC08 | TTCAGGGAACAAACCAAGTTACGT | 75164 | FAK47171 |
| SL26 | run1 | BC19 | GTTCCTCGTGCAGTGTCAAGAGAT | 65675 | FAK47171 |
| SL76 | run1 | BC20 | TTGCGTCCTGTTACGAGAACTCAT | 67020 | FAK47171 |
| SL27 | run1 | BC31 | TGCGTACAGCAATCAGTTACATTG | 127831 | FAK47171 |
| SL77 | run1 | BC32 | CCAGTAGAAGTCCGACAACGTCAT | 49056 | FAK47171 |
| SL28 | run1 | BC43 | CTTTCGTTGTTGACTCGACGGTAG | 86020 | FAK47171 |
| SL79 | run1 | BC44 | AGTAGAAAGGGTTCCTTCCCACTC | 104474 | FAK47171 |
| SL29 | run1 | BC55 | TGGAAGATGAGACCCTGATCTACG | 48176 | FAK47171 |
| SL30 | run1 | BC67 | GTTTGTCATACTCGTGTGCTCACC | 120285 | FAK47171 |
| SL68 | run1 | BC79 | TCTCGGAGATAGTTCTCACTGCTG | 144453 | FAK47171 |
| SL74 | run1 | BC91 | GGCTCCATAGGAACTCACGCTACT | 104551 | FAK47171 |
| SL5 | run2 | BC09 | AACTAGGCACAGCGAGTCTTGGTT | 138049 | FAK54862 |
| SL7 | run2 | BC21 | GAGCCTCTCATTGTCCGTTCTCTA | 137257 | FAK54862 |
| SL11 | run2 | BC33 | CAGACTTGGTACGGTTGGGTAACT | 147067 | FAK54862 |
| SL12 | run2 | BC45 | GATCCAACAGAGATGCCTTCAGTG | 181957 | FAK54862 |
| SL20 | run2 | BC56 | TCACTACTCAACAGGTGGCATGAA | 161970 | FAK54862 |
| SL14 | run2 | BC57 | GCTAGGTCAATCTCCTTCGGAAGT | 128650 | FAK54862 |
| SL50 | run2 | BC68 | GAATCTAAGCAAACACGAAGGTGG | 241108 | FAK54862 |
| SL16 | run2 | BC69 | TACAGTCCGAGCCTCATGTGATCT | 128248 | FAK54862 |
| SL57 | run2 | BC80 | CGGATGAACATAGGATAGCGATTC | 191114 | FAK54862 |
| SL17 | run2 | BC81 | CCTCATCTTGTGAAGTTGTTTCGG | 124104 | FAK54862 |
| SL59 | run2 | BC92 | TTGTGAGTGGAAAGATACAGGACC | 190822 | FAK54862 |
| SL19 | run2 | BC93 | AGTTTCCATCACTTCAGACTTGGG | 171065 | FAK54862 |
| SL61 | run3 | BC10 | AAGCGTTGAAACCTTTGTCCTCTC | 54284 | FAK54862 |
| SL8 | run3 | BC11 | GTTTCATCTATCGGAGGGAATGGA | 139614 | FAK54862 |
| SL63 | run3 | BC22 | ACCACTGCCATGTATCAAAGTACG | 61746 | FAK54862 |
| SL9 | run3 | BC23 | CTTACTACCCAGTGAACCTCCTCG | 86683 | FAK54862 |
| SL71 | run3 | BC34 | GGACGAAGAACTCAAGTCAAAGGC | 107061 | FAK54862 |
| SL13 | run3 | BC35 | CTACTTACGAAGCTGAGGGACTGC | 94172 | FAK54862 |
| SL73 | run3 | BC46 | GCTGTGTTCCACTTCATTCTCCTG | 101645 | FAK54862 |
| SL23 | run3 | BC47 | GTGCAACTTTCCCACAGGTAGTTC | 223611 | FAK54862 |
| SL78 | run3 | BC58 | CAGGTTACTCCTCCGTGAGTCTGA | 113182 | FAK54862 |
| SL2 | run3 | BC70 | ACCGAGATCCTACGAATGGAGTGT | 86534 | FAK54862 |
| SL3 | run3 | BC82 | ACGGTATGTCGAGTTCCAGGACTA | 80197 | FAK54862 |
| SL6 | run3 | BC94 | GATTGTCCTCAAACTGCCACCTAC | 103463 | FAK54862 |
| SL4 | run4 | BC12 | CAGGTAGAAAGAAGCAGAATCGGA | 303732 | FAK53681 |
| SL24 | run4 | BC24 | GCATAGTTCTGCATGATGGGTTAG | 194671 | FAK53681 |
| SL49 | run4 | BC36 | ATGTCCCAGTTAGAGGAGGAAACA | 278836 | FAK53681 |
| SL51 | run4 | BC48 | CATCTGGAACGTGGTACACCTGTA | 308489 | FAK53681 |
| SL72 | run4 | BC59 | TCAATCAAGAAGGGAAAGCAAGGT | 369691 | FAK53681 |
| SL56 | run4 | BC60 | CATGTTCAACCAAGGCTTCTATGG | 299769 | FAK53681 |
| SL65 | run4 | BC71 | CCTGGGAGCATCAGGTAGTAACAG | 196888 | FAK53681 |
| SL60 | run4 | BC72 | TAGCTGACTGTCTTCCATACCGAC | 340940 | FAK53681 |
| SL80 | run4 | BC83 | TGGCTTGATCTAGGTAAGGTCGAA | 592506 | FAK53681 |
| SL67 | run4 | BC84 | GTAGTGGACCTAGAACCTGTGCCA | 258466 | FAK53681 |
| SL18 | run4 | BC95 | CCTGTCTGGAAGAAGAATGGACTT | 202089 | FAK53681 |
| SL69 | run4 | BC96 | CTGAACGGTCATAGAGTCCACCAT | 407953 | FAK53681 |

**Supplementary Table S3.** Results of genus enrichment analyses to determine which host tree group the tested genera were more likely to occur in. Quasi-Poisson GLMs were fit for genera ordered by abundance after rarefaction (to 1000 reads in the Taylor and Tedersoo, and to 164 reads per sample in the ONT dataset), to estimate abundance after rarefaction in each host group. All 58 samples were included in both of the Illumina datasets, while the ONT dataset consisted of 30 samples. The proportion for estimates of gymno- vs. angiosperms was calculated as exponential coefficient of the model ‘rarefied abundance ~ host group’ and indicated the enrichment in either host tree group. Taxa and reads for each host group represent values before rarefaction. Only results for genera resulting in significant (*P*<0.05) differences between host tree groups are shown.

| **Order** | **Method** | **Genus** | **Proportion gymno/**  **angio estimate** | **Taxa in Angio** | **Reads in Angio** | **Taxa in Gymno** | **Reads in Gymno** | ***P*-value** | **Host group** |
| --- | --- | --- | --- | --- | --- | --- | --- | --- | --- |
| 2 | Taylor | *Hormonema* | 4044.625 | 1 | 1 | 8 | 4978 | 9.136e-07 | Gymno |
| 5 | Taylor | *Aspergillus* | 8.167 | 17 | 274 | 15 | 2754 | 0.02 | Gymno |
| 6 | Taylor | *Plagiostoma* | 2.8e-09 | 6 | 2101 | 0 | 0 | 2.8e-08 | Angio |
| 7 | Taylor | *Cladosporium* | 0.118 | 6 | 1989 | 4 | 288 | 0.001 | Angio |
| 9 | Taylor | *Ramichloridium* | 94194335 | 0 | 0 | 6 | 1852 | 0.007 | Gymno |
| 10 | Taylor | *Gnomoniopsis* | 4.199e-09 | 8 | 1400 | 0 | 0 | 1.226e-06 | Angio |
| 11 | Taylor | *Diplodia* | 139.181 | 1 | 10 | 1 | 1713 | 0.031 | Gymno |
| 12 | Taylor | *Colletotrichum* | 0.001 | 8 | 1386 | 1 | 1 | 7.765e-06 | Angio |
| 13 | Taylor | *Apiognomonia* | 5.049e-09 | 1 | 1164 | 0 | 0 | 5.322e-05 | Angio |
| 1 | Tedersoo |  | 2.572 | 189 | 18153 | 2024 | 57473 | 3.743e-21 | Gymno |
| 2 | Tedersoo | unidentified | 0.009 | 46 | 7931 | 28 | 88 | 1.897e-06 | Angio |
| 3 | Tedersoo | *Penicillium* | 0.063 | 42 | 3549 | 11 | 277 | 8.271e-06 | Angio |
| 4 | Tedersoo | *Cladosporium* | 0.006 | 7 | 2783 | 3 | 21 | 5.333e-09 | Angio |
| 5 | Tedersoo | *Plagiostoma* | 0.004 | 4 | 2501 | 2 | 13 | 2.511e-07 | Angio |
| 6 | Tedersoo | *Gnomoniopsis* | 8.245e-07 | 6 | 2470 | 1 | 1 | 8.244e-07 | Angio |
| 7 | Tedersoo | *Colletotrichum* | 5.379e-06 | 9 | 2417 | 1 | 1 | 5.379e-06 | Angio |
| 2 | ONT | *Aspergillus* | 17.530 | 35 | 39 | 233 | 293 | 3.428e-05 | Gymno |
| 6 | ONT | *Colletotrichum* | 2.037e-08 | 203 | 233 | 0 | 0 | 0.049 | Angio |

**Supplementary Table S4.** Numbers of OTUs and genera (indicated in brackets) assigned to different fungal lifestyles and those not assigned to a lifestyle, for the three metabarcoding datasets, and the relative abundances (in percent) of each lifestyle group of the total fungal OTUs assigned at genus level. The number of total and fungal OTUs of each method is indicated at the top for each dataset.

| ***Total number of OTUs***  ***Fungal OTUs*** | | **Taylor**  ***n=1391***  ***n=1224*** | **Tedersoo**  ***n=3598***  ***n=809*** | **ONT**  ***n=14094***  ***n=12761*** |
| --- | --- | --- | --- | --- |
| Total OTUs assigned to genus,  of those: | | *n*=999 (244 gen.) | *n*=608 (217 gen.) | *n*=9668 (226 gen.) |
|  | Not assigned to lifestyle (none of the below) | 519 (69 gen.)  37% | 235 (62 gen.)  35.4% | 4047 (72 gen.)  42.7% |
|  | Plant endophytes | 17 (5 gen.)  2.8% | 11 (5 gen.)  3.6% | 538 (4 gen.)  2.6% |
|  | Plant pathogens | 284 (85 gen.)  41.4% | 225 (79 gen.)  40.4% | 3844 (82 gen.)  42% |
|  | Plant saprotrophs | 179 (85 gen.)  18.8% | 137 (71 gen.)  20.6% | 1239 (68 gen.)  12.7% |

**Supplementary Table S5.** Results of Pairwise PERMANOVAs testing the differences in community composition of the seed mycobiota between combinations of dataset (n=3) and host group (n=2), resulting in 15 pairs. The results are based on genus-level community matrices for which the robust Aitchisons distances were calculated. The method used for *P*-value adjustment was ‘fdr’.

| **Comparison pairs** | **Df** | **Sums Of Squares** | **F** | **R^2^** | ***P*-value** | **P-adjusted** | **Sign.** |
| --- | --- | --- | --- | --- | --- | --- | --- |
| Taylor Gymnosperms vs Taylor Angiosperms | 1 | 1035.769 | 5.030 | 0.082 | 0.001 | 0.00136 | * |
| Taylor Gymnosperms vs Tedersoo Gymnosperms | 1 | 89.787 | 0.799 | 0.013 | 0.732 | 0.78429 |  |
| Taylor Gymnosperms vs Tedersoo Angiosperms | 1 | 892.952 | 4.477 | 0.074 | 0.001 | 0.00136 | * |
| Taylor Gymnosperms vs ONT Gymnosperms | 1 | 236.069 | 2.646 | 0.045 | 0.001 | 0.00136 | * |
| Taylor Gymnosperms vs ONT Angiosperms | 1 | 280.752 | 2.616 | 0.049 | 0.001 | 0.00136 | * |
| Taylor Angiosperms vs Tedersoo Gymnosperms | 1 | 1009.356 | 6.219 | 0.100 | 0.001 | 0.00136 | * |
| Taylor Angiosperms vs Tedersoo Angiosperms | 1 | 107.186 | 0.403 | 0.008 | 0.999 | 0.99900 |  |
| Taylor Angiosperms vs ONT Gymnosperms | 1 | 760.021 | 5.339 | 0.096 | 0.001 | 0.00136 | * |
| Taylor Angiosperms vs ONT Angiosperms | 1 | 483.648 | 2.866 | 0.060 | 0.001 | 0.00136 | * |
| Tedersoo Gymnosperms vs Tedersoo Angiosperms | 1 | 837.787 | 5.375 | 0.088 | 0.001 | 0.00136 | * |
| Tedersoo Gymnosperms vs ONT Gymnosperms | 1 | 101.055 | 2.215 | 0.038 | 0.004 | 0.00462 | * |
| Tedersoo Gymnosperms vs ONT Angiosperms | 1 | 187.319 | 3.151 | 0.058 | 0.001 | 0.00136 | * |
| Tedersoo Angiosperms vs ONT Gymnosperms | 1 | 611.147 | 4.523 | 0.083 | 0.001 | 0.00136 | * |
| Tedersoo Angiosperms vs ONT Angiosperms | 1 | 373.374 | 2.323 | 0.049 | 0.002 | 0.00250 | * |
| ONT Gymnosperms vs ONT Angiosperms | 1 | 82.913 | 3.520 | 0.073 | 0.001 | 0.00136 | * |

## References

Chao, A., Wang, Y. T., & Jost, L. (2013). Entropy and the species accumulation curve: a novel entropy estimator via discovery rates of new species. *Methods in Ecology and Evolution*, *4*(11), 1091–1100. https://doi.org/10.1111/2041-210X.12108

Jost, L. (2006). Entropy and diversity. *Oikos*, *113*(2), 363–375. https://doi.org/10.1111/j.2006.0030-1299.14714.x

Oksanen, J. (2022). *Vegan: ecological diversity*.
